# Extensive sampling and thorough taxonomic assessment of Afrotropical Rhyssinae (Hymenoptera, Ichneumonidae) reveals two new species and demonstrates the limitations of previous sampling efforts

**DOI:** 10.3897/zookeys.878.37845

**Published:** 2019-10-07

**Authors:** Tapani Hopkins, Heikki Roininen, Simon van Noort, Gavin R. Broad, Kari Kaunisto, Ilari E. Sääksjärvi

**Affiliations:** 1 Zoological Museum, Biodiversity Unit, FI-20014 University of Turku, Finland University of Turku Turku Finland; 2 Department of Environmental and Biological Sciences, University of Eastern Finland, Joensuu, Finland University of Eastern Finland Joensuu Finland; 3 Department of Research & Exhibitions, South African Museum, Iziko Museums of South Africa, PO Box 61, Cape Town 8000, South Africa Iziko Museums of South Africa Cape Town South Africa; 4 Department of Biological Sciences, University of Cape Town, Private Bag, Rondebosch, 7701, South Africa University of Cape Town Cape Town South Africa; 5 Department of Life Sciences, the Natural History Museum, London SW7 5BD, UK Natural History Museum London United Kingdom

**Keywords:** Africa, Ichneumonidae, Kibale National Park, Uganda Malaise trapping 2014–2015

## Abstract

Tropical forest invertebrates, such as the parasitoid wasp family Ichneumonidae, are poorly known. This work reports some of the first results of an extensive survey implemented in Kibale National Park, Uganda. A total of 456 individuals was caught of the subfamily Rhyssinae Morley, 1913, which in the Afrotropical region was previously known from only 30 specimens. Here, the six species found at the site are described and the Afrotropical Rhyssinae are reviewed. Two new species, *Epirhyssa johanna* Hopkins, **sp. nov.** and *E. quagga***sp. nov.**, are described and a key, diagnostic characters, and descriptions for all 13 known Afrotropical species are provided, including the first description of the male of *Epirhyssa overlaeti* Seyrig, 1937. *Epirhyssa gavinbroadi* Rousse & van Noort, 2014, **syn. nov.** is proposed to be a synonym of *E. uelensis* Benoit, 1951. Extensive sampling with Malaise traps gave an unprecedented sample size, and the method is recommended for other poorly known tropical areas.

## Introduction

Like many taxa, the parasitoid wasps of the family Ichneumonidae are poorly known in the tropics. So much so, that the family was once assumed to be exceptionally species-poor in the equatorial region and to peak in species richness in mid latitudes (Owen and Owen 1974, Janzen and Pond 1975, but see Morrison et al. 1979). More recent extensive sampling has revealed rich ichneumonid faunas in the tropical forests of Costa Rica and Amazonia (Gauld 1991, Gaston and Gauld 1993, Sääksjärvi et al. 2004), and the family appears to be too poorly sampled in the tropics to allow reliable conclusions as to the distribution of its species richness (Quicke 2012). In Sub-Saharan Africa, only 2097 of an estimated 12,100 species have been described (Townes and Townes 1973, Yu et al. 2016, van Noort 2019), and even sites that have been studied remain strongly under-sampled (e.g., van Noort et al. 2000, van Noort 2004, Hopkins et al. 2018).

The ichneumonid subfamily Rhyssinae is especially poorly known in the Afrotropical region. It was reviewed in 2014 (Rousse and van Noort 2014), resulting in five new species and a total of twelve species for Sub-Saharan Africa. This is very low compared to the currently known (and increasing) 49 Neotropical, 125 Oriental, 23 Australasian, 40 Palaearctic, and 17 Nearctic species (Yu et al. 2016). Insufficient sampling, rather than a genuine scarcity, is presumably the main reason for the low African species count: even after gathering together material from several African and European museums, Rousse and van Noort (2014) were unable to find more than 30 specimens collected from the whole of the Afrotropical region.

One possible reason for the lack of rhyssine specimens is that adequately inventorying tropical ichneumonids appears to need long-term, extensive sampling (for reasons summarised in Hopkins et al. 2019b). Such sampling is laborious and has rarely been done in the tropics. Ichneumonids have been extensively sampled by Malaise traps in Costa Rica (e.g., more than 1200 trap months: Gauld 1991, about 190 trap months: Shapiro and Pickering 2000) and in Amazonia (185 trap months: Sääksjärvi et al. 2004, at least 72 additional trap months: Gómez et al. 2017). For Sub-Saharan Africa, we know of only two large-scale sampling programs, one of which returned an unexpectedly low sample size of ichneumonids (231 trap months using traps smaller than the standard size, Hopkins et al. 2018). The results of the second program have not yet been published (258 trap months conducted by SvN in Hantam National Botanical Garden, South Africa).

In this and a sister paper, we report the first results of an extensive one-year sampling of Afrotropical ichneumonids in Kibale National Park, Uganda. In the sister paper we report the ecological results for the subfamily Rhyssinae, including descriptions of the habitat use and phenology of the species (Hopkins et al. 2019b). Here, we update the taxonomy of the subfamily and describe the new species found at our site, providing a key, diagnostic characters, and updates to descriptions for all known Afrotropical Rhyssinae.

## Materials and methods

We sampled ichneumonids with 34 Malaise traps for a full year (2014–2015) in Kibale National Park, Uganda. The traps were placed in a wide variety of habitats ranging from primary forest to clear-cut former plantations and farmland, and the total sampling effort was roughly 382 trap months (11662.13 trap days, of which 271.16 trap days were unrepresentative of a normal catch). We describe the sampling in greater detail in Hopkins et al. (2019b) and its associated dataset (Hopkins et al. 2019a). As well as using Malaise traps, we also collected ichneumonids by hand netting. Hand-netted ichneumonids were stored individually in 96% ethanol.

We processed the samples at the Zoological Museum of the University of Turku, Finland. We separated the ichneumonoid wasps (families Ichneumonidae and Braconidae) from the Malaise samples, then pinned the subfamily Rhyssinae and sorted specimens into species. We did not pin the hand netted rhyssines; instead, we stored these specimens individually in 96% ethanol. The samples are deposited at the Zoological Museum.

Layer photographs were taken using a Canon 7 D mark 2 digital camera, attached to an Olympus SZX 16 stereomicroscope. Photographs were captured using the programmes Deep Focus 3.1 and Quick Photo Camera 2.3. Photographs were finally combined with the program Zerene and edited in Photoshop CC. Additional images were acquired at SAMC with a Leica LAS 4.9 imaging system, comprising a Leica Z16 microscope (using either a 2 × or 5 × objective) with a Leica DFC450 Camera and 0.63 × video objective attached. The imaging process, using an automated Z-stepper, was managed using the Leica Application Suite V 4.9 software installed on a desktop computer. Diffused lighting was achieved using a Leica LED5000 HDI dome. All images presented in this paper, as well as supplementary images, are available at www.waspweb.org.

**Figure 1.**
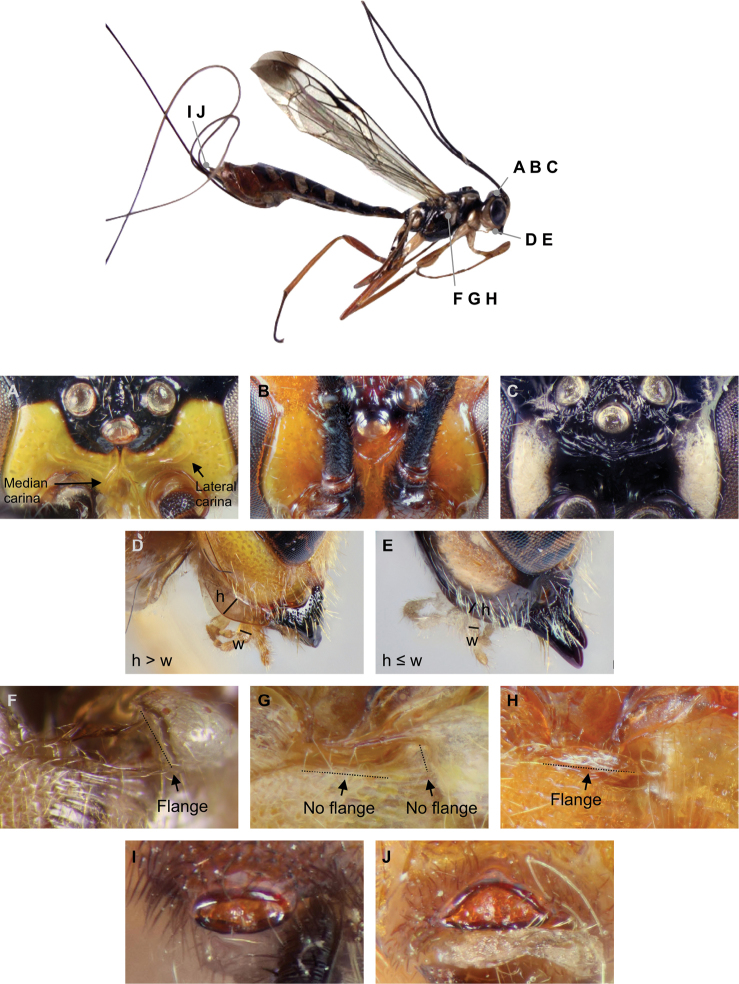
Diagnostic characters for the thirteen known Afrotropical rhyssine species. The figure shows the frons median carinae (**A** converge, **B** diverge, **C** absent), frons lateral carinae (**A** present, **B** absent), hypostomal flange (**D** wider than second maxillary palp, **E** narrower than or comparable to second maxillary palp), subalar prominence (**F** flanged, **G** no flange), mesopleuron margin (**H** flanged, **G** no flange) and female apical horn (**I** ellipse, **J** half-ellipse). Not shown are the epicnemial carina (laterally absent, only just reaches the mesopleuron, reaches high onto the mesopleuron), areolet (present, absent) and tergite 3 structure (mostly striate, mostly punctate, mostly smooth). The images are of http://mus.utu.fi/ZMUT.5766, http://mus.utu.fi/ZMUT.2520 (**A, F**), http://mus.utu.fi/ZMUT.5853 (**B, D, H, J**), http://mus.utu.fi/ZMUT.5788 (**C, E, I**) and http://mus.utu.fi/ZMUT.5663 (**G**). Image **F** has been flipped horizontally.

Because earlier diagnostic characters (Rousse and van Noort 2014) did not work well with our material, we collected a partly new set of nine diagnostic characters for all Afrotropical species (Fig. 1). We retrieved diagnostic characters for the previously known 30 specimens from Rousse and van Noort (2014) if available, or from the specimens themselves if not.

Morphological terminology largely follows Gauld (1991) and body measurements follow Rousse and van Noort (2014). We measured body length from the base of the antennae to the tip of the metasoma. If the metasoma was bent, we measured it in several line segments. We measured the slenderness of tergite 1 by dividing the median length by the apical width. The images used to take the measurements are available in the supplementary material (Hopkins et al. 2019c).

We generated an identification key automatically, based on the nine diagnostic characters (Hopkins et al. 2019c). The diagnostic characters, descriptions, identification key and data for all Ugandan specimens are available in table format in the supplementary material (Hopkins et al. 2019c).

### Repositories

**MNHN**Muséum national d’Histoire naturelle, Paris, France (Agnièle Touret-Alby)

**NHMUK**Natural History Museum, London, UK (Gavin Broad)

**NMSA**KwaZulu-Natal Museum, Pietermaritzburg, South Africa (John Midgley)

**RMCA**Royal Museum for Central Africa, Tervueren, Belgium (Stéphane Hanot)

**SAMC**Iziko South African Museum, Cape Town, South Africa (Simon van Noort)

**ZMUT**Zoological Museum of the University of Turku, Finland (Ilari Sääksjärvi)

## Results

We caught 448 rhyssines by Malaise sampling and eight by hand netting. They belonged to six species of which two are new. We provide a key to all 13 known Afrotropical species below. We also provide diagnostic characters and descriptions or updates to descriptions for all species. The key, diagnostic characters, and descriptions are also available in table form in the supplementary material (Hopkins et al. 2019c). Online identification keys are available on www.waspweb.org.

### Key to species

**Table:** 

1	Fore wing with a closed areolet (Fig. 2A).[Black and orange species with infuscate wings; Fig. 72]	***Megarhyssa babaulti* Seyrig, 1937**
–	Fore wing without an areolet (Fig. 2B)	**2**
2	Subalar prominence with a lateral flange (Fig. 2C).[Yellow species with black markings; Fig. 57]	***Epirhyssa uelensis* Benoit, 1951**
–	Subalar prominence without a lateral flange (Fig. 2D)	**3**
3	Dorsal margin of mesopleuron with a raised flange (Fig. 2D)	**4**
–	Dorsal margin of mesopleuron without a raised flange (Fig. 2E)	**5**
4	Apical horn of metasoma shaped like an ellipse in posterior view (Fig. 2F), frons with median carinae that converge before the ocelli, frons with lateral carinae. [Red, yellow, and black species; Fig. 32]	***Epirhyssa overlaeti* Seyrig, 1937**
–	Apical horn of metasoma shaped like a half-ellipse in posterior view (Fig. 2G), frons with median carinae that diverge before continuing towards the lateral ocelli, frons without lateral carinae. [Orange species, sometimes with black markings; Fig. 7]	***Epirhyssa ghesquierei* Seyrig, 1937**
5	Epicnemial carina laterally absent, does not reach mesopleuron	**6**
–	Epicnemial carina present on mesopleuron	**7**
6	Frons with median carinae that diverge before continuing towards the lateral ocelli (cf. Fig. 1B). [Yellow species with black on metasoma; Fig. 28]	***Epirhyssa migratoria* Seyrig, 1932**
–	Frons smooth, without median carinae (Fig. 2H). [Predominantly orange species; Fig. 14]	***Epirhyssa johanna* sp. nov.**
7	Frons with median carinae that diverge before continuing towards the lateral ocelli (cf. Fig. 1B), frons with lateral carinae. [Black and orange species with infuscate wings; Fig. 68]	***Epirhyssa villemantae* Rousse & van Noort, 2014**
–	Frons with median carinae that converge before the ocelli or without median carinae (Fig. 2I), frons without lateral carinae (Fig. 2I)	**8**
8	Tergite 3 densely striate (Fig. 2J), frons striate but without median carinae. [Black, white and orange species; Fig. 39]	***Epirhyssa quagga* sp. nov.**
–	Tergite 3 mostly smooth or mostly punctate (Fig. 2K), frons with median carinae	**9**
9	Tergite 3 mostly smooth	**10**
–	Tergite 3 mostly punctate, over 50% of surface (Fig. 2K)	**11**
10	Epicnemial carina only just reaches the mesopleuron. [Hypostomal carina raised into a low flange, yellow-orange species with yellow and infuscate wings; Fig. 25]	***Epirhyssa maynei* Benoit, 1952**
–	Epicnemial carina long, reaches the approximate height of the mesopleural pit. [Hypostomal carina raised into an elevated flange, yellow species with black mesosternum; Fig. 21]	***Epirhyssa leroyi* Benoit, 1951**
11	Hypostomal carina raised into an elevated flange (Fig. 2L, see also Fig. 1D). [Black and white species; Fig. 3]	***Epirhyssa brianfisheri* Rousse & van Noort, 2014**
–	Hypostomal carina raised into a low flange (Fig. 2M, see also Fig. 1E)	**12**
12	Apical horn of metasoma shaped like a half-ellipse in posterior view (Fig. 2N). [Yellow species with black markings as in Fig. 50]	***Epirhyssa tombeaodiba* Rousse & van Noort, 2014**
–	Apical horn of metasoma shaped like an ellipse in posterior view (Fig. 2O). [Yellow species with black markings as in Fig. 46]	***Epirhyssa shaka* Rousse & van Noort, 2014**

**Figure 2.**
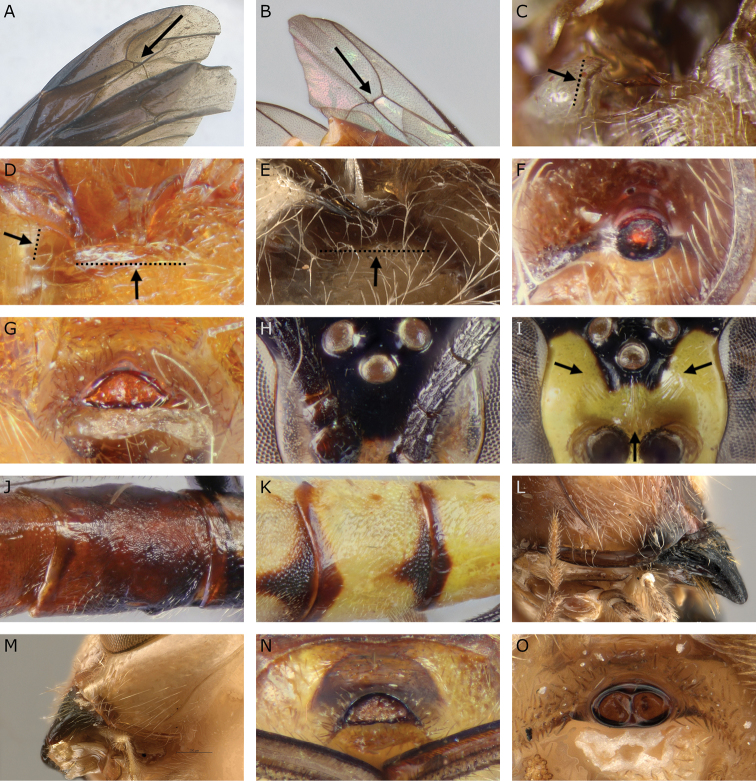
Diagnostic character traits used in the identification key. **A** Areolet (*Megarhyssa babaulti* holotype) **B** open areolet (http://mus.utu.fi/ZMUT.5788) **C** subalar prominence flange (http://mus.utu.fi/ZMUT.2520) **D** subalar prominence without flange and dorsal margin of mesopleuron with flange (http://mus.utu.fi/ZMUT.5853) **E** dorsal margin of mesopleuron without flange (SAMC SAM–HYM–P048018) **F** elliptical apical horn of metasoma (http://mus.utu.fi/ZMUT.5766) **G** half-elliptical apical horn of metasoma (http://mus.utu.fi/ZMUT.5853) **H** frons without carinae (http://mus.utu.fi/ZMUT.4920) **I** frons without lateral carinae (http://mus.utu.fi/ZMUT.3234) **J** striate tergite 3 (http://mus.utu.fi/ZMUT.5788) **K** punctate tergite 3 (http://mus.utu.fi/ZMUT.5663) **L** hypostomal carina raised into elevated flange (SAMC SAM–HYM–P048018) **M** hypostomal carina raised into low flange (*E. shaka* holotype) **N** half-elliptical apical horn of metasoma (http://mus.utu.fi/ZMUT.5663) **O** elliptical apical horn of metasoma (*E. shaka* holotype). Image **A** is from van Noort (2019). Image **D** has been flipped horizontally.

### Taxonomic descriptions

#### Family Ichneumonidae Latreille, 1802

**Subfamily Rhyssinae Morley, 1913**

**Diagnosis.** The subfamily Rhyssinae can be recognised by a combination of the transverse rugae covering much of the mesoscutum; the short, broad mandibles and small, rectangular clypeus; the long ovipositor; and the female 8^th^ metasomal tergite being produced posteriorly as a truncate horn-like projection. Other genera that present a potential confusion risk, such as *Pseudorhyssa* Merril (Pimplinae), *Certonotus* Kriechbaumer, and *Apechoneura* Kriechbaumer (Labeninae) are not present in the Afrotropical region.

##### Genus Epirhyssa

Taxon classificationAnimaliaHymenopteraIchneumonidae

Cresson, 1865

36652094-E3B6-55B9-B3BF-D4189A6283D8

Hierax Tosquinet, 1903: 255.
Rhyssonota Kriechbaumer, 1890: 489.
Sychnostigma Baltazar, 1961: 75.

###### Diagnosis.

The genus *Epirhyssa* is easily recognised in the Afrotropical region as the species lack the fore wing areolet (vein 3rs-m is missing), whereas the areolet is closed by vein 3rs-m in *Megarhyssa* Ashmead, the only other rhyssine genus found in the Afrotropical region.

*Epirhyssa* can be distinguished from other rhyssine genera by the lack of an areolet (cf. *Rhyssella* Rohwer, *Lytarmes* Cameron), the lack of an anterior glymma on tergite 1 (cf. *Rhyssa* Gravenhorst), the upper tooth being slightly wider than the lower and not subdivided (cf. *Triancyra* Baltazar, *Myllenyxis* Baltazar) and the pterostigma being angled where it meets the metacarpus (compared to gradually merging in *Cyrtorhyssa* Baltazar) (Baltazar 1964, Townes 1969, Porter 2001). Old World *Epirhyssa* have fore wing vein 2rs-m only a little proximal to 2m-cu, unlike New World species. The species are rather heterogeneous, with confusion with other genera particularly likely in the Oriental region, and the genus may well prove not to be monophyletic.

###### Distribution.

**Afrotropical region**: Central African Republic, Cameroon, Democratic Republic of Congo, Madagascar, Nigeria, South Africa, Uganda.

**Australasian region**: Papua New Guinea.

**Nearctic region**: Mexico, U.S.A.

**Neotropical region**: Argentina, Brazil, Bolivia, Costa Rica, Cuba, Ecuador, Guatemala, Guyana, Nicaragua, Paraguay, Peru, Trinidad.

**Oriental region**: China, India, Indonesia, Japan, Malaysia, Myanmar, Philippines, Singapore, Vietnam.

**Palaearctic region**: Russia.

##### Epirhyssa brianfisheri

Taxon classificationAnimaliaHymenopteraIchneumonidae

Rousse & van Noort, 2014

9B22C444-85EB-5839-BE3C-08D09E62A5CF

Figs 3–6

###### Material examined.

**Type material**: CENTRAL AFRICAN REPUBLIC:

• 1 ♀, holotype; Préfecture Sangha-Mbaéré, Réserve Spéciale de Forêt Dense de Dzanga-Sangha (12.7 km, 326 degrees NW of Bayanga); 3°00.27'N, 16°11.55'E; alt. 420 m; 13 May 2001; Simon van Noort leg.; Sweep; CAR01–S158; Lowland rainforest; SAMC SAM–HYM–P048018.

**Known material**: One specimen (1 ♀, see Rousse and van Noort 2014, data above).

###### Diagnosis.

This species can be distinguished from other Afrotropical Rhyssinae by the combination of an elevated hypostomal flange, the absence of a raised flange on the dorsal margin of the mesopleuron, an elliptical apical horn of the metasoma, and a finely punctate (over 50% of surface) tergite 3. In practice its colour pattern makes it instantly recognisable.

***Head***: frons with median carinae converging before continuing towards median ocellus, without lateral carinae; hypostomal carina raised into an elevated flange, its height greater than the maximum width of the second maxillary palp segment.

***Mesosoma***: subalar prominence without a lateral flange; mesopleuron without a flange along the dorsal margin; epicnemial carina reaches the approximate height of the mesopleural pit.

***Metasoma***: tip of apical horn elliptical in posterior view; tergite 3 punctate.

###### Distribution.

Central African Republic.

**Figures 3–6.**
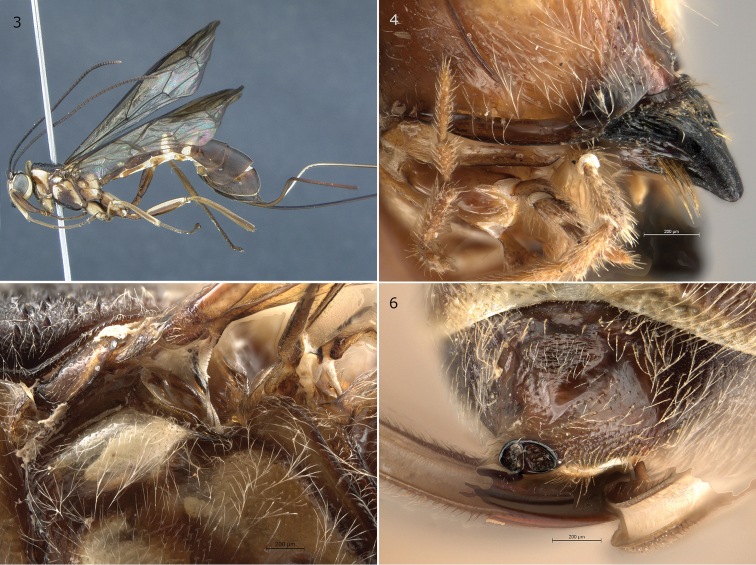
*Epirhyssa brianfisheri* female (holotype, SAM–HYM–P048018). This species was not found in Uganda. **3** Habitus **4** hypostomal flange **5** mesopleuron dorsal margin **6** apical horn of metasoma. Figure **3** is from van Noort (2019).

##### Epirhyssa ghesquierei

Taxon classificationAnimaliaHymenopteraIchneumonidae

Seyrig, 1937

9EE7D460-6856-5D03-A2FB-1940A7F18BB7

Figs 7–13

###### Material examined.

**Type material**: DEMOCRATIC REPUBLIC OF CONGO:

• 1 ♀, holotype; Eala [0°4.22'N, 18°18.15'E]; Nov. 1935; “J. Ghesquière”; “R. Dét. G 3330”; RMCARMCA-ENT-000017927

• 1 ♂, paratype; Bambesa; Dec. 1946; “P.L. Benoit”; RMCARMCA-ENT-000017928.

**Non-type material**: CAMEROON:

• 1 ♂; Korup; Dec. 1980–Jan. 1981; Mrs D. Jackson leg.; NHMUK

• 1 ♂; Korup; 1981; Mrs D. Jackson leg.; NHMUK.

UGANDA:

• 1 ♀; Kibale National Park, Kanyawara, Site R93, Malaise trap R93T1; 0.5653N, 30.3568E (WGS84); alt. 1510 m (GPS, WGS84); 23 Sep. 2014–7 Oct. 2014; Tapani Hopkins leg.; ZMUThttp://mus.utu.fi/ZMUT.53

• 1 ♀; same data as preceding; Site K30S, Malaise trap K30ST4; 0.5414N, 30.3755E (WGS84); alt. 1420 m (GPS, WGS84); 25 Aug. 2015–11 Sep. 2015; ZMUThttp://mus.utu.fi/ZMUT.1263

• 1 ♀; same data as preceding; Site K15, Malaise trap K15T2; 0.5843N, 30.3644E (WGS84); alt. 1470 m (GPS, WGS84); 4 May 2015–20 May 2015; ZMUThttp://mus.utu.fi/ZMUT.5592

• 1 ♀; same data as preceding; Site CC, Malaise trap CCT1; 0.5497N, 30.3673E (WGS84); alt. 1450 m (GPS, WGS84); 15 Dec. 2014–29 Dec. 2014; ZMUThttp://mus.utu.fi/ZMUT.5701

• 1 ♀; same data as preceding; 24 Feb. 2015–10 Mar. 2015; ZMUThttp://mus.utu.fi/ZMUT.5737

• 1 ♀; same data as preceding; 13 Jan. 2015–27 Jan. 2015; ZMUThttp://mus.utu.fi/ZMUT.5853

• 1 ♂; Kibale National Park, Kanyawara, Site K31, Malaise trap K31T4; 0.5362N, 30.3486E (WGS84); alt. 1460 m (GPS, WGS84); 29 Dec. 2014–16 Jan. 2015; Tapani Hopkins leg.; ZMUThttp://mus.utu.fi/ZMUT.2300

• 1 ♂; same data as preceding; Site CC, Malaise trap CCT1; 0.5497N, 30.3673E (WGS84); alt. 1450 m (GPS, WGS84); 30 Jun. 2015–14 Jul. 2015; ZMUThttp://mus.utu.fi/ZMUT.5610.

**Non-type material** (only diagnostic characters checked): UGANDA:

• 109 ♀; Kibale National Park, Kanyawara; Tapani Hopkins leg.; ZMUThttp://mus.utu.fi/ZMUT.1250, http://mus.utu.fi/ZMUT.1251, http://mus.utu.fi/ZMUT.1252, http://mus.utu.fi/ZMUT.1257, http://mus.utu.fi/ZMUT.1258, http://mus.utu.fi/ZMUT.1260, http://mus.utu.fi/ZMUT.1264, http://mus.utu.fi/ZMUT.1269, http://mus.utu.fi/ZMUT.1271, http://mus.utu.fi/ZMUT.1282, http://mus.utu.fi/ZMUT.1335, http://mus.utu.fi/ZMUT.1365, http://mus.utu.fi/ZMUT.1526, http://mus.utu.fi/ZMUT.1695, http://mus.utu.fi/ZMUT.1720, http://mus.utu.fi/ZMUT.2569, http://mus.utu.fi/ZMUT.2799, http://mus.utu.fi/ZMUT.3010, http://mus.utu.fi/ZMUT.3077, http://mus.utu.fi/ZMUT.3095, http://mus.utu.fi/ZMUT.3100, http://mus.utu.fi/ZMUT.3103, http://mus.utu.fi/ZMUT.3104, http://mus.utu.fi/ZMUT.3459, http://mus.utu.fi/ZMUT.3495, http://mus.utu.fi/ZMUT.3496, http://mus.utu.fi/ZMUT.3529, http://mus.utu.fi/ZMUT.3542, http://mus.utu.fi/ZMUT.3611, http://mus.utu.fi/ZMUT.3638, http://mus.utu.fi/ZMUT.4375, http://mus.utu.fi/ZMUT.4738, http://mus.utu.fi/ZMUT.5594, http://mus.utu.fi/ZMUT.5598, http://mus.utu.fi/ZMUT.5599, http://mus.utu.fi/ZMUT.5603, http://mus.utu.fi/ZMUT.5612, http://mus.utu.fi/ZMUT.5615, http://mus.utu.fi/ZMUT.5617, http://mus.utu.fi/ZMUT.5621, http://mus.utu.fi/ZMUT.5624, http://mus.utu.fi/ZMUT.5627, http://mus.utu.fi/ZMUT.5634, http://mus.utu.fi/ZMUT.5636, http://mus.utu.fi/ZMUT.5638, http://mus.utu.fi/ZMUT.5639, http://mus.utu.fi/ZMUT.5649, http://mus.utu.fi/ZMUT.5654, http://mus.utu.fi/ZMUT.5659, http://mus.utu.fi/ZMUT.5661, http://mus.utu.fi/ZMUT.5664, http://mus.utu.fi/ZMUT.5666, http://mus.utu.fi/ZMUT.5667, http://mus.utu.fi/ZMUT.5671, http://mus.utu.fi/ZMUT.5676, http://mus.utu.fi/ZMUT.5677, http://mus.utu.fi/ZMUT.5680, http://mus.utu.fi/ZMUT.5685, http://mus.utu.fi/ZMUT.5686, http://mus.utu.fi/ZMUT.5691, http://mus.utu.fi/ZMUT.5695, http://mus.utu.fi/ZMUT.5696, http://mus.utu.fi/ZMUT.5706, http://mus.utu.fi/ZMUT.5707, http://mus.utu.fi/ZMUT.5710, http://mus.utu.fi/ZMUT.5711, http://mus.utu.fi/ZMUT.5715, http://mus.utu.fi/ZMUT.5716, http://mus.utu.fi/ZMUT.5718, http://mus.utu.fi/ZMUT.5721, http://mus.utu.fi/ZMUT.5723, http://mus.utu.fi/ZMUT.5724, http://mus.utu.fi/ZMUT.5735, http://mus.utu.fi/ZMUT.5745, http://mus.utu.fi/ZMUT.5747, http://mus.utu.fi/ZMUT.5751, http://mus.utu.fi/ZMUT.5753, http://mus.utu.fi/ZMUT.5756, http://mus.utu.fi/ZMUT.5759, http://mus.utu.fi/ZMUT.5760, http://mus.utu.fi/ZMUT.5761, http://mus.utu.fi/ZMUT.5769, http://mus.utu.fi/ZMUT.5770, http://mus.utu.fi/ZMUT.5776, http://mus.utu.fi/ZMUT.5782, http://mus.utu.fi/ZMUT.5786, http://mus.utu.fi/ZMUT.5796, http://mus.utu.fi/ZMUT.5799, http://mus.utu.fi/ZMUT.5800, http://mus.utu.fi/ZMUT.5802, http://mus.utu.fi/ZMUT.5809, http://mus.utu.fi/ZMUT.5810, http://mus.utu.fi/ZMUT.5812, http://mus.utu.fi/ZMUT.5813, http://mus.utu.fi/ZMUT.5819, http://mus.utu.fi/ZMUT.5826, http://mus.utu.fi/ZMUT.5827, http://mus.utu.fi/ZMUT.5830, http://mus.utu.fi/ZMUT.5837, http://mus.utu.fi/ZMUT.5840, http://mus.utu.fi/ZMUT.5841, http://mus.utu.fi/ZMUT.5842, http://mus.utu.fi/ZMUT.5843, http://mus.utu.fi/ZMUT.5847, http://mus.utu.fi/ZMUT.6015, http://mus.utu.fi/ZMUT.6043, http://mus.utu.fi/ZMUT.6050, http://mus.utu.fi/ZMUT.6051, http://mus.utu.fi/ZMUT.6053

• 47 ♂; Kibale National Park, Kanyawara; Tapani Hopkins leg.; ZMUThttp://mus.utu.fi/ZMUT.1351, http://mus.utu.fi/ZMUT.1359, http://mus.utu.fi/ZMUT.1588, http://mus.utu.fi/ZMUT.1791, http://mus.utu.fi/ZMUT.2170, http://mus.utu.fi/ZMUT.2505, http://mus.utu.fi/ZMUT.2622, http://mus.utu.fi/ZMUT.2647, http://mus.utu.fi/ZMUT.3098, http://mus.utu.fi/ZMUT.3099, http://mus.utu.fi/ZMUT.3528, http://mus.utu.fi/ZMUT.3684, http://mus.utu.fi/ZMUT.3694, http://mus.utu.fi/ZMUT.4322, http://mus.utu.fi/ZMUT.4514, http://mus.utu.fi/ZMUT.4636, http://mus.utu.fi/ZMUT.4641, http://mus.utu.fi/ZMUT.5588, http://mus.utu.fi/ZMUT.5590, http://mus.utu.fi/ZMUT.5602, http://mus.utu.fi/ZMUT.5611, http://mus.utu.fi/ZMUT.5633, http://mus.utu.fi/ZMUT.5651, http://mus.utu.fi/ZMUT.5656, http://mus.utu.fi/ZMUT.5660, http://mus.utu.fi/ZMUT.5682, http://mus.utu.fi/ZMUT.5700, http://mus.utu.fi/ZMUT.5703, http://mus.utu.fi/ZMUT.5714, http://mus.utu.fi/ZMUT.5730, http://mus.utu.fi/ZMUT.5731, http://mus.utu.fi/ZMUT.5744, http://mus.utu.fi/ZMUT.5746, http://mus.utu.fi/ZMUT.5777, http://mus.utu.fi/ZMUT.5779, http://mus.utu.fi/ZMUT.5795, http://mus.utu.fi/ZMUT.5828, http://mus.utu.fi/ZMUT.5833, http://mus.utu.fi/ZMUT.5848, http://mus.utu.fi/ZMUT.5852, http://mus.utu.fi/ZMUT.5859, http://mus.utu.fi/ZMUT.6046, http://mus.utu.fi/ZMUT.6047, http://mus.utu.fi/ZMUT.6048, http://mus.utu.fi/ZMUT.6049, http://mus.utu.fi/ZMUT.6052, http://mus.utu.fi/ZMUT.6054.

**Known material**: 168 specimens (164 Ugandan, 4 other):

112 ♀, 44 ♂; Ugandan specimens caught by Malaise trap, data above and also in supplementary material (Hopkins et al. 2019c).

3 ♀, 5 ♂; Ugandan hand-netted specimens, data above and also in supplementary material (Hopkins et al. 2019c).

1 ♀, 3 ♂; see Rousse and van Noort (2014), data above in material examined.

###### Diagnosis.

This species can be distinguished from other Afrotropical Rhyssinae by the combination of a half-elliptical apical horn of the metasoma and a mostly smooth tergite 3.

***Head***: frons with diverging median carinae, without clear lateral carinae; hypostomal carina raised into an elevated flange, its height greater than the maximum width of the second maxillary palp segment.

***Mesosoma***: subalar prominence without a lateral flange; mesopleuron with an elevated flange along the dorsal margin; epicnemial carina reaches the approximate height of the mesopleural pit.

***Metasoma***: tip of apical horn half-elliptical in posterior view; tergite 3 mostly smooth.

###### Additional or updated characters.

Apart from the diagnosis, we provide the following additional or updated character traits to the description in Rousse and van Noort (2014).

**Female.**

Body length 11.4 mm–17.2 mm. Frons rugulose or smooth, often with more or less distinct rugae that fan out from the median carinae towards the ocelli. Antenna with 32–34 flagellar segments. Tergites mostly smooth, but with variable structure on some tergites (4–7 pubescent and anterior margins of 3–5 slightly punctate or striate in Ugandan specimens, 3–6 shallowly punctate with anterior striations in other specimens), tergite 1 2.2–2.5 times as long as apically wide. The Ugandan specimens are more orange than yellow in colour, generally have no dark spots on the lateral lobes of the mesoscutum, and the colour of their interocellar area varies from orange (most frequent) to black.

**Male.** Body length 11.5 mm–14.1 mm. Antenna with 31–33 flagellar segments. Anterior margin of tergite 3 sometimes neither punctate nor striate. Tergite 1 2.5–3.0 times as long as apically wide. Males are smaller than females on average.

###### Distribution.

Democratic Republic of Congo, Cameroon. New record: Uganda.

###### Biology.

In Uganda, this species was most abundantly caught in primary forest near decaying wood, during the dry season (Hopkins et al. 2019b). It has not been caught outside the forest. Many of the hand-netted individuals were caught after landing on a fallen tree trunk (*Uvariopsis congensis* Robyns & Ghesq.). The males especially seemed to be repeatedly visiting the tree.

**Figures 7–13.**
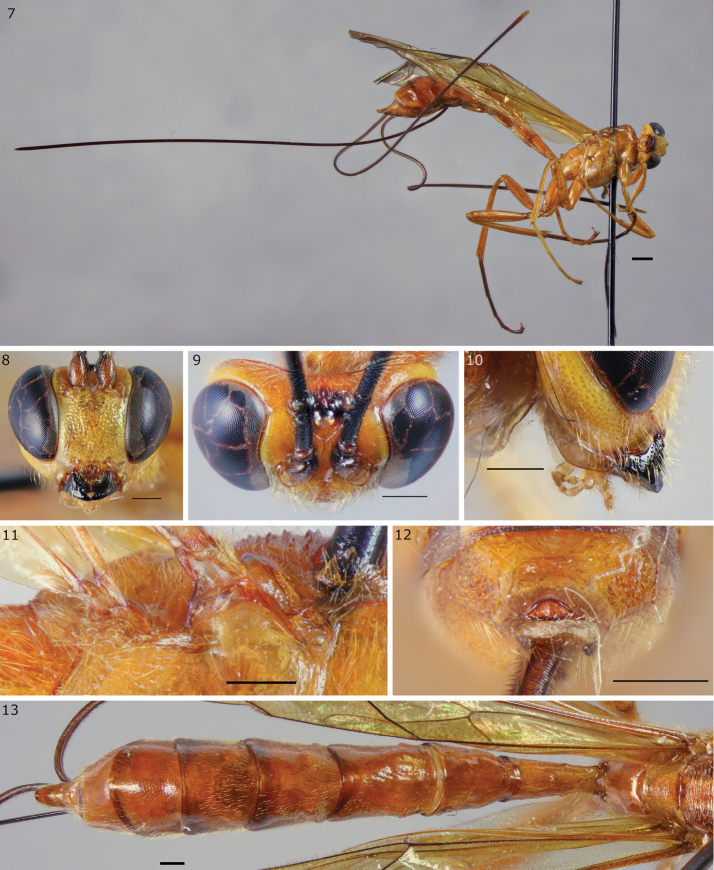
*Epirhyssa ghesquierei* female (http://mus.utu.fi/ZMUT.5853), a species found in Uganda. **7** Habitus **8** face and clypeus **9** frons **10** hypostomal flange **11** mesopleuron dorsal margin **12** apical horn of metasoma **13** tergites 1–7. Scale bars: 0.5 mm (**8–13**), 1 mm (**7**).

##### Epirhyssa johanna

Taxon classificationAnimaliaHymenopteraIchneumonidae

Hopkins sp. nov.

35061147-23AF-5725-8208-155D6A1A3118

http://zoobank.org/67413463-3BD1-4812-B6F5-DA6E768ABE03

Figs 14–20

###### Material examined.

**Type material**: UGANDA:

• 1 ♀, holotype; Kibale National Park, Kanyawara, Site R03, Malaise trap R03T2; 0.5403N, 30.3608E (WGS84); alt. 1490 m (GPS, WGS84); 7 May 2015–21 May 2015; Tapani Hopkins leg.; ZMUThttp://mus.utu.fi/ZMUT.4920.

**Known material**: One specimen (1 ♀, Ugandan specimen, data above).

###### Diagnosis.

This species can be distinguished from other Afrotropical Rhyssinae by the combination of a smooth frons without median carinae and a laterally absent epicnemial carina. No other species has the same colour pattern.

***Head***: frons without median carinae, without lateral carinae; hypostomal carina raised into a low flange, its height slightly less than or equivalent to the maximum width of the second maxillary palp segment.

***Mesosoma***: subalar prominence without a lateral flange; mesopleuron without a flange along the dorsal margin; epicnemial carina laterally absent.

***Metasoma***: tip of apical horn elliptical in posterior view; tergite 3 mostly smooth.

###### Description (female).

Body length 8.4 mm.

***Head***: Frons without median carinae, without lateral carinae, smooth or very faintly striate. Occipital carina interrupted dorsally and interrupted or extremely faint near hypostomal carina. Hypostomal carina raised into a low flange, its height slightly less than or equivalent to maximum width of second maxillary palp segment. Face smooth or very faintly punctate. Clypeus sparsely punctate, with a median apical tubercle. Antenna with 28 flagellar segments.

***Mesosoma***: Subalar prominence without a lateral flange. Mesopleuron without a flange along the dorsal margin. Epicnemial carina laterally absent. Fore wing with 2m-cu distal to rs-m.

***Metasoma***: Tip of apical horn elliptical (flattened ellipse) in posterior view. Tergites mostly smooth, anterior of tergites 2–6 medially striate, tergite 1 1.2 times as long as apically wide.

***Colour***: General colour orange. Other colour: white face, lower 1/4 of genae, lateral frons, black mandibles, median frons, occiput, upper genae, and median spot on apical half of tergite 6 and entire tergite 7, dark brown hind tarsi. Antennae black. Ovipositor sheaths dark testaceous. Wings hyaline, faintly infuscate near apex.

**Male.** Unknown.

###### Etymology.

Dedicated to Johanna Hopkins, the first author’s wife. This species is known from only one, quite exceptional, female specimen.

###### Distribution.

Uganda.

###### Remarks.

Only one specimen was caught during 382 trap months of sampling, in a habitat (successional forest logged 2002–2004) that generally yielded few rhyssines.

**Figures 14–20.**
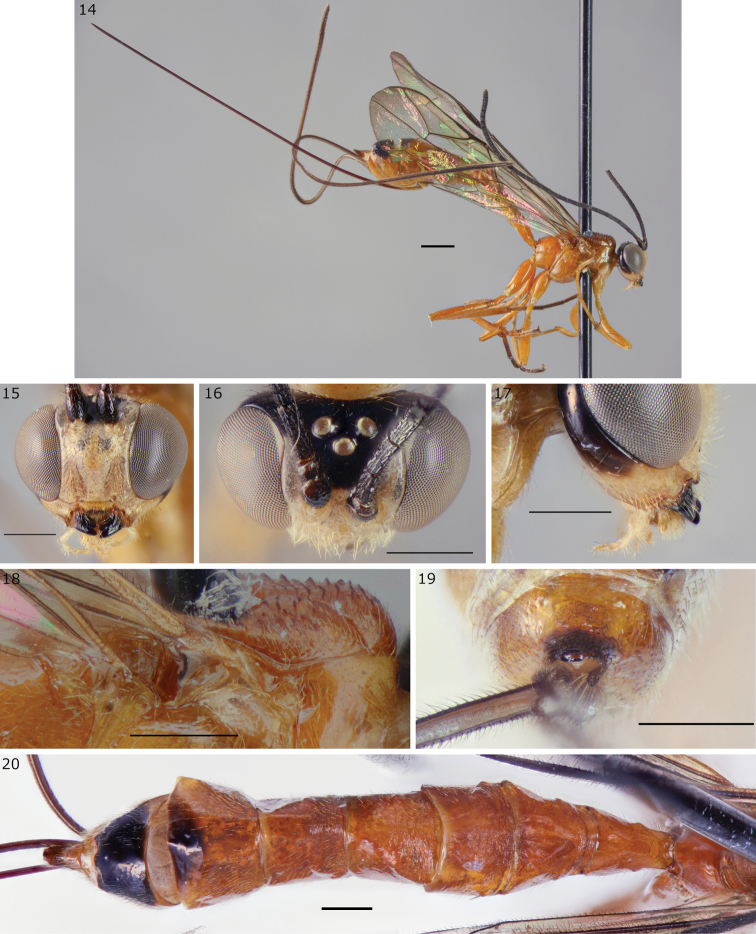
*Epirhyssa johanna* female (holotype http://mus.utu.fi/ZMUT.4920), a new species from Uganda. **14** habitus **15** face and clypeus **16** frons **17** hypostomal flange **18** mesopleuron dorsal margin **19** apical horn of metasoma **20** tergites 1–7. Scale bars: 0.5 mm (**15–20**), 1 mm (**14**).

##### Epirhyssa leroyi

Taxon classificationAnimaliaHymenopteraIchneumonidae

Benoit, 1951

66E92BAF-B28F-5DCC-AE05-8C219A9C55BE

Figs 21–24

###### Material examined.

**Type material**: DEMOCRATIC REPUBLIC OF CONGO:

• 1 ♀, holotype; Bambesa [03°28'N 25°43'E]; Dec. 1933; “J.V. Leroy”; RMCARMCA-ENT-000017923

• 1 ♀, paratype; Ubangui-Bumba; Dec. 1939; H. de Saeger; RMCARMCA-ENT-000017924.

**Known material**: Two specimens (2 ♀, see Rousse and van Noort 2014, data above).

###### Diagnosis.

This species can be distinguished from other Afrotropical Rhyssinae by the combination of converging median carinae on the frons, the absence of lateral carinae on the frons, an epicnemial carina that reaches high onto the mesopleuron, and a mostly smooth tergite 3. No other species is known to have a black mesosternum.

***Head***: frons with median carinae converging before continuing towards median ocellus, without lateral carinae; hypostomal carina raised into an elevated flange, its height slightly greater than the maximum width of the second maxillary palp segment.

***Mesosoma***: subalar prominence without a lateral flange; mesopleuron without a flange along the dorsal margin; epicnemial carina reaches the approximate height of the mesopleural pit.

***Metasoma***: tip of apical horn elliptical in posterior view; tergite 3 mostly smooth.

###### Distribution.

Democratic Republic of Congo.

**Figures 21–24.**
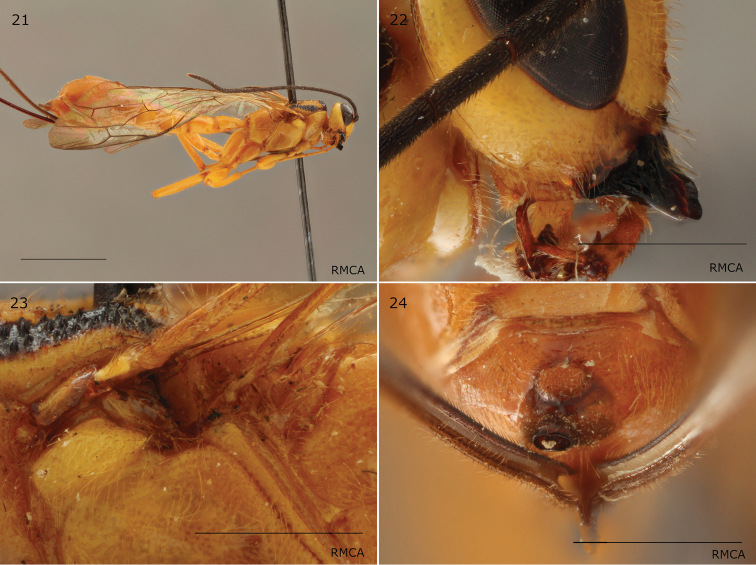
*Epirhyssa leroyi* female (paratype RMCA-ENT-000017924). This species was not found in Uganda. **21** Habitus **22** hypostomal flange **23** mesopleuron dorsal margin **24** apical horn of metasoma. Scale bars 1 mm (**22–24**), 5 mm (**21**). Figures courtesy of RMCA (Stéphane Hanot).

##### Epirhyssa maynei

Taxon classificationAnimaliaHymenopteraIchneumonidae

Benoit, 1952

45CF9BA7-12CB-5EC0-A793-D2F010E23EE9

Figs 25–27

###### Material examined.

**Non-type material**: CENTRAL AFRICAN REPUBLIC:

• 1 ♂; Préfecture Sangha-Mbaéré, Parc National de Dzanga-Ndoki (38.6 km, 173 degrees S of Lidjombo); 2°21.60'N, 16°09.20'E; alt. 350 m; 27 May 2001; Simon van Noort leg.; Handnet; CAR01-H25; Lowland rainforest; SAMC SAM–HYM–P049437.

**Known material**: Three specimens (0 Ugandan, 3 other):

1 ♂, holotype; see Rousse and van Noort (2014); Democratic Republic of Congo, Albertville [Kalemie, 05°56'N, 29°12'E]; Jul. 1918; “R. Mayné”; MRAC.

1 ♂, paratype; see Rousse and van Noort (2014); Democratic Republic of Congo, Bambesa; Dec. 1946; “P.L. Benoit”; MRAC.

1 ♂; see Rousse and van Noort (2014), data above in material examined.

###### Diagnosis.

This species can be distinguished from other Afrotropical Rhyssinae by the epicnemial carina only just reaching the mesopleuron. The fore wing colour pattern makes it instantly recognisable.

***Head***: frons with median carinae converging before continuing towards median ocellus, without lateral carinae; hypostomal carina raised into a low flange, its height slightly less than or equivalent to the maximum width of the second maxillary palp segment.

***Mesosoma***: subalar prominence without a lateral flange; mesopleuron without a flange along the dorsal margin; epicnemial carina short, barely extending onto mesopleuron.

***Metasoma***: tip of apical horn of unknown shape (no females are known); tergite 3 mostly smooth.

###### Distribution.

Democratic Republic of Congo, Central African Republic.

**Figures 25–27.**
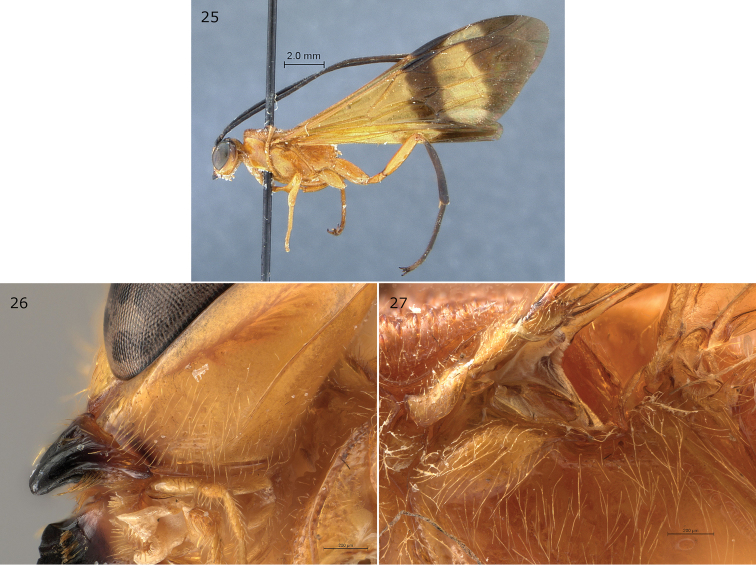
*Epirhyssa maynei* male (**25**: holotype, **26–27**: SAM–HYM–P049437). This species was not found in Uganda. **25** Habitus **26** hypostomal flange **27** mesopleuron dorsal margin. Figure **25** is from van Noort (2019).

##### Epirhyssa migratoria

Taxon classificationAnimaliaHymenopteraIchneumonidae

Seyrig, 1932

7D7269AD-B092-5787-A07F-A800D5E1FF5D

Figs 28–31

###### Material examined.

**Type material**: MADAGASCAR:

• 1 ♀, paralectotype; Rogez, Forêt Cote Est; Mar. 1932; “A Seyrig”; NHMUK.

**Known material**: Eight specimens (0 Ugandan, 8 other):

1 ♀, lectotype; see Rousse and van Noort (2014); Madagascar, Rogez [18°18'S, 48°32'E]; Sep. 1930; “A. Seyrig”; MNHN EY8815.

3 ♂; see Rousse and van Noort (2014), same label data as lectotype.

1 ♀, paralectotype; previously unpublished specimen, data above in material examined.

1 ♀, 2 ♂; previously unpublished specimens, same data as paralectotype except collection dates Apr. 1931, Sep. 1932, Dec. 1932; NHMUK.

###### Diagnosis.

This species can be distinguished from other Afrotropical Rhyssinae by the combination of diverging median carinae on the frons, the lateral absence of the epicnemial carina, and an open areolet. Its yellow and black colour pattern is distinctive.

***Head***: frons with median carinae diverging towards ocelli, without lateral carinae; hypostomal carina raised into a low flange, its height slightly less than or equivalent to the maximum width of the second maxillary palp segment.

***Mesosoma***: subalar prominence without a lateral flange; mesopleuron without a flange along the dorsal margin; epicnemial carina laterally absent.

***Metasoma***: tip of apical horn elliptical in posterior view; tergite 3 mostly smooth.

###### Distribution.

Madagascar.

**Figures 28–31.**
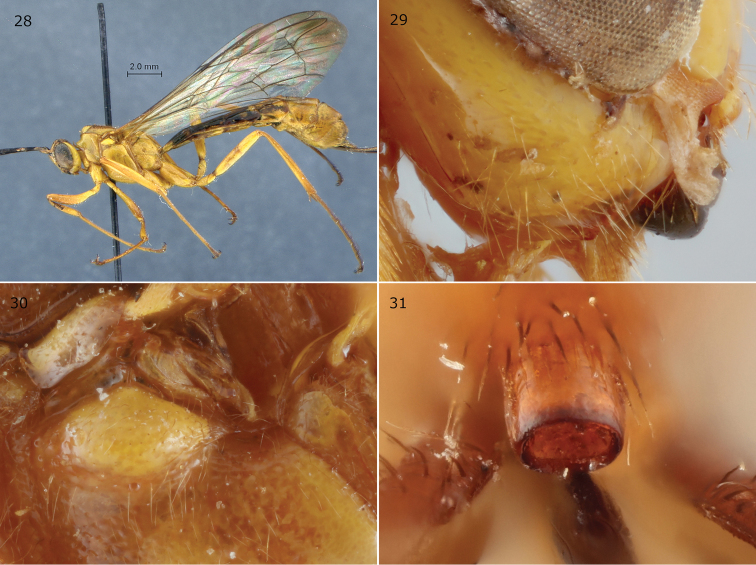
*Epirhyssa migratoria* female (holotype). This species was not found in Uganda. **28** Habitus **29** hypostomal flange **30** mesopleuron dorsal margin **31** apical horn of metasoma. Figure **28** is from van Noort (2019).

##### Epirhyssa overlaeti

Taxon classificationAnimaliaHymenopteraIchneumonidae

Seyrig, 1937

89669B5F-6792-54AF-830F-660F2CE3E525

Figs 32–38

###### Material examined.

**Non-type material**: UGANDA:

• 1 ♀; Kibale National Park, Kanyawara, Site CC, Malaise trap CCT1; 0.5497N, 30.3673E (WGS84); alt. 1450 m (GPS, WGS84); 10 Mar. 2015–24 Mar. 2015; Tapani Hopkins leg.; ZMUThttp://mus.utu.fi/ZMUT.1249

• 1 ♀; same data as preceding; Site K31, Malaise trap K31T4; 0.5362N, 30.3486E (WGS84); alt. 1460 m (GPS, WGS84); 16 Jul. 2015–30 Jul. 2015; ZMUThttp://mus.utu.fi/ZMUT.2291

• 1 ♀; same data as preceding; Site CC, Malaise trap CCT1; 0.5497N, 30.3673E (WGS84); alt. 1450 m (GPS, WGS84); 18 Nov. 2014–2 Dec. 2014; ZMUThttp://mus.utu.fi/ZMUT.5591

• 1 ♀; same data as preceding; Site R93, Malaise trap R93T2; 0.5654N, 30.3593E (WGS84); alt. 1510 m (GPS, WGS84); 20 May 2015–1 Jun. 2015; ZMUThttp://mus.utu.fi/ZMUT.5766

• 1 ♀; same data as preceding; Site HILL, Malaise trap HILLT1; 0.5486N, 30.3614E (WGS84); alt. 1520 m (GPS, WGS84); 30 Jul. 2015–13 Aug. 2015; ZMUThttp://mus.utu.fi/ZMUT.6013

• 1 ♂; Kibale National Park, Kanyawara, Site K31, Malaise trap K31T2; 0.5427N, 30.3482E (WGS84); alt. 1460 m (GPS, WGS84); 9 Apr. 2015–23 Apr. 2015; Tapani Hopkins leg.; ZMUThttp://mus.utu.fi/ZMUT.1280

• 1 ♂; same data as preceding; Site CC, Malaise trap CCT1; 0.5497N, 30.3673E (WGS84); alt. 1450 m (GPS, WGS84); 24 Feb. 2015–10 Mar. 2015; ZMUThttp://mus.utu.fi/ZMUT.5738

• 1 ♂; same data as preceding; Site K31, Malaise trap K31T3; 0.5360N, 30.3469E (WGS84); alt. 1450 m (GPS, WGS84); 26 Aug. 2015–12 Sep. 2015; ZMUThttp://mus.utu.fi/ZMUT.5792.

**Non-type material** (only diagnostic characters checked): UGANDA:

• 56 ♀; Kibale National Park, Kanyawara; Tapani Hopkins leg.; ZMUThttp://mus.utu.fi/ZMUT.1261, http://mus.utu.fi/ZMUT.1262, http://mus.utu.fi/ZMUT.1270, http://mus.utu.fi/ZMUT.1336, http://mus.utu.fi/ZMUT.1337, http://mus.utu.fi/ZMUT.1338, http://mus.utu.fi/ZMUT.1345, http://mus.utu.fi/ZMUT.1364, http://mus.utu.fi/ZMUT.1721, http://mus.utu.fi/ZMUT.1761, http://mus.utu.fi/ZMUT.1762, http://mus.utu.fi/ZMUT.1820, http://mus.utu.fi/ZMUT.2056, http://mus.utu.fi/ZMUT.2058, http://mus.utu.fi/ZMUT.2161, http://mus.utu.fi/ZMUT.2706, http://mus.utu.fi/ZMUT.2724, http://mus.utu.fi/ZMUT.2856, http://mus.utu.fi/ZMUT.2985, http://mus.utu.fi/ZMUT.3080, http://mus.utu.fi/ZMUT.3092, http://mus.utu.fi/ZMUT.3541, http://mus.utu.fi/ZMUT.3737, http://mus.utu.fi/ZMUT.5595, http://mus.utu.fi/ZMUT.5600, http://mus.utu.fi/ZMUT.5626, http://mus.utu.fi/ZMUT.5630, http://mus.utu.fi/ZMUT.5640, http://mus.utu.fi/ZMUT.5645, http://mus.utu.fi/ZMUT.5658, http://mus.utu.fi/ZMUT.5672, http://mus.utu.fi/ZMUT.5674, http://mus.utu.fi/ZMUT.5675, http://mus.utu.fi/ZMUT.5697, http://mus.utu.fi/ZMUT.5704, http://mus.utu.fi/ZMUT.5720, http://mus.utu.fi/ZMUT.5727, http://mus.utu.fi/ZMUT.5728, http://mus.utu.fi/ZMUT.5729, http://mus.utu.fi/ZMUT.5741, http://mus.utu.fi/ZMUT.5742, http://mus.utu.fi/ZMUT.5749, http://mus.utu.fi/ZMUT.5752, http://mus.utu.fi/ZMUT.5773, http://mus.utu.fi/ZMUT.5781, http://mus.utu.fi/ZMUT.5783, http://mus.utu.fi/ZMUT.5784, http://mus.utu.fi/ZMUT.5789, http://mus.utu.fi/ZMUT.5791, http://mus.utu.fi/ZMUT.5793, http://mus.utu.fi/ZMUT.5798, http://mus.utu.fi/ZMUT.5814, http://mus.utu.fi/ZMUT.5815, http://mus.utu.fi/ZMUT.5818, http://mus.utu.fi/ZMUT.5822, http://mus.utu.fi/ZMUT.5839

• 14 ♂; Kibale National Park, Kanyawara; Tapani Hopkins leg.; ZMUThttp://mus.utu.fi/ZMUT.2906, http://mus.utu.fi/ZMUT.3086, http://mus.utu.fi/ZMUT.3089, http://mus.utu.fi/ZMUT.3642, http://mus.utu.fi/ZMUT.5622, http://mus.utu.fi/ZMUT.5652, http://mus.utu.fi/ZMUT.5758, http://mus.utu.fi/ZMUT.5767, http://mus.utu.fi/ZMUT.5771, http://mus.utu.fi/ZMUT.5785, http://mus.utu.fi/ZMUT.5790, http://mus.utu.fi/ZMUT.5832, http://mus.utu.fi/ZMUT.5838, http://mus.utu.fi/ZMUT.5877

• 1 unknown sex; Kibale National Park, Kanyawara; Tapani Hopkins leg.; ZMUThttp://mus.utu.fi/ZMUT.6045.

**Known material**: 81 specimens (79 Ugandan, 2 other):

61 ♀, 17 ♂, 1 U; Ugandan specimens, data above and also in supplementary material (Hopkins et al. 2019c).

1 ♀, holotype; see Rousse and van Noort (2014); Democratic Republic of Congo, Lulua [10°37'S 24°54'E], Kapanga; Apr. 1933; “F.G. Overlaet”; “R. Dét”; MRAC “F 3330”.

1 ♀; see Rousse and van Noort (2014); Cameroon, Nkoemvon; Jul. 1980-Aug. 1980; “Ms. D. Jackson”; NHMUK.

**Figures 32–38.**
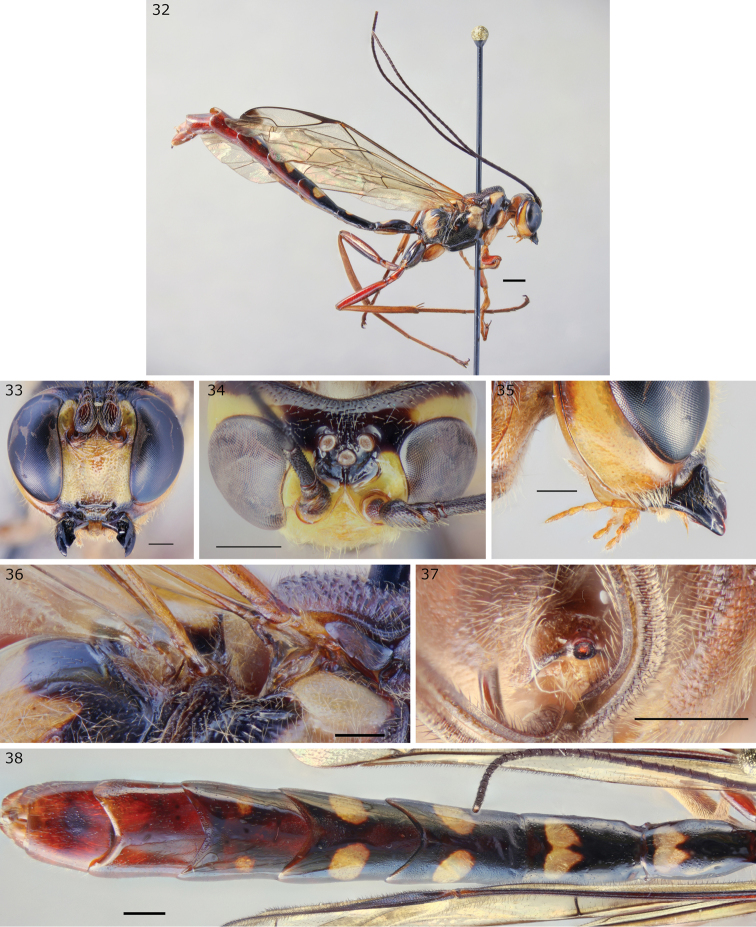
*Epirhyssa overlaeti* male (http://mus.utu.fi/ZMUT.1280) and female (**34**: http://mus.utu.fi/ZMUT.2291, **37**: http://mus.utu.fi/ZMUT.5766), a species found in Uganda. **32** Habitus **33** face and clypeus **34** frons **35** hypostomal flange **36** mesopleuron dorsal margin **37** apical horn of metasoma **38** tergites 1–7. Scale bars: 0.5 mm (**33–38**), 1 mm (**32**).

###### Diagnosis.

This species can be distinguished from other Afrotropical Rhyssinae by the combination of converging median carinae on the frons, lateral carinae on the frons, and the absence of a lateral flange on the subalar prominence. In practice its unique colour pattern and large size make it instantly recognisable.

***Head***: frons with strong median carinae converging before continuing towards median ocellus, with lateral carinae curving towards lateral ocelli; hypostomal carina raised into an elevated flange, its height slightly greater than the maximum width of the second maxillary palp segment.

***Mesosoma***: subalar prominence without a lateral flange; mesopleuron with a raised flange along the dorsal margin; epicnemial carina reaches the approximate height of the mesopleural pit.

***Metasoma***: tip of apical horn almost circular in posterior view; tergite 3 mostly smooth.

###### Description (male).

Body length 16.4 mm–25.1 mm. Males seem slightly smaller than females on average.

***Head***: Frons with strong median carinae converging before continuing towards median ocellus, with lateral carinae curving towards lateral ocelli, often with faint traces of lateral rugae. Occipital carina interrupted dorsally. Hypostomal carina raised into an elevated flange, its height slightly greater than maximum width of second maxillary palp segment. Face punctate. Clypeus punctate, without a clear median apical tubercle. Antenna with 38–40 flagellar segments.

***Mesosoma***: Subalar prominence without a lateral flange. Mesopleuron with a raised flange along dorsal margin. Epicnemial carina reaches approximate height of mesopleural pit. Fore wing with 2m-cu distal to rs-m.

***Metasoma***: Tergites 1–5 mostly smooth, 6–7 smooth or sparsely pubescent, tergite 1 1.8–2.2 times as long as apically wide.

***Colour***: General colour mottled dark testaceous, testaceous and pale yellow, pale yellow patches of tergites 1 and 2 are fused (cf. female). Antennae black. Wings hyaline, infuscate near apex.

**Additional or updated characters.** Apart from the diagnosis and description of the male, we provide the following additional or updated character traits to the description in Rousse and van Noort (2014).

**Female.** Body length 14.7 mm–37.8 mm. Frons often with faint traces of lateral rugae. Face punctate, transversely rugulose-punctate or transversely striate. Antenna with 38–43 flagellar segments (38–41 in Ugandan specimens). Tergites 1–5 mostly smooth, 6–7 often pubescent, anterior margins of tergites 3–6 (sometimes only 4–5) sightly striate or punctate, tergite 1 1.4–1.8 times as long as apically wide. The colour patches vary in extent, with the pale yellow patches of tergite 1 fused in small individuals.

###### Distribution.

Democratic Republic of Congo, Cameroon. New record: Uganda.

###### Biology.

In Uganda, this species was mostly caught in primary forest near decaying wood (Hopkins et al. 2019b). It has not been caught outside the forest.

###### Remarks.

*Epirhyssa overlaeti* was earlier known from only two females. We describe the male for the first time.

##### Epirhyssa quagga sp. nov.

Taxon classificationAnimaliaHymenopteraIchneumonidae

5CA38040-F440-5B6D-928F-FCD9E282FED5

http://zoobank.org/9BABD7C8-7644-45BA-9985-96408F898402

Figs 39–45

###### Material examined.

**Type material**: UGANDA:

• 1 ♀, holotype; Kibale National Park, Kanyawara, Site CC, Malaise trap CCT1; 0.5497N, 30.3673E (WGS84); alt. 1450 m (GPS, WGS84); 11 Aug. 2015–25 Aug. 2015; Tapani Hopkins leg.; ZMUThttp://mus.utu.fi/ZMUT.5788

• 1 ♀, paratype; same data as preceding; Site R03, Malaise trap R03T2; 0.5403N, 30.3608E (WGS84); alt. 1490 m (GPS, WGS84); 21 May 2015–4 Jun. 2015; ZMUThttp://mus.utu.fi/ZMUT.1500

• 1 ♀, paratype; same data as preceding; Site K31, Malaise trap K31T3; 0.5360N, 30.3469E (WGS84); alt. 1450 m (GPS, WGS84); 23 May 2015–4 Jun. 2015; ZMUThttp://mus.utu.fi/ZMUT.4238

• 1 ♀, paratype; same data as preceding; Site K30S, Malaise trap K30ST4; 0.5414N, 30.3755E (WGS84); alt. 1420 m (GPS, WGS84); 2 Dec. 2014–15 Dec. 2014; ZMUThttp://mus.utu.fi/ZMUT.5743

• 1 ♀, paratype; same data as preceding; Site R01, Malaise trap R01T2; 0.5501N, 30.3561E (WGS84); alt. 1600 m (GPS, WGS84); 27 Mar. 2015–10 Apr. 2015; ZMUThttp://mus.utu.fi/ZMUT.5801

• 1 ♂, paratype; Kibale National Park, Kanyawara, Site CC, Malaise trap CCT1; 0.5497N, 30.3673E (WGS84); alt. 1450 m (GPS, WGS84); 2 Jun. 2015–17 Jun. 2015; Tapani Hopkins leg.; ZMUThttp://mus.utu.fi/ZMUT.3074

• 1 ♂, paratype; same data as preceding; Site K30S, Malaise trap K30ST2; 0.5392N, 30.3771E (WGS84); alt. 1480 m (GPS, WGS84); 5 May 2015–19 May 2015; ZMUThttp://mus.utu.fi/ZMUT.4000

• 1 ♂, paratype; same data as preceding; Site K15, Malaise trap K15T4; 0.5845N, 30.3674E (WGS84); alt. 1510 m (GPS, WGS84); 4 May 2015–20 May 2015; ZMUThttp://mus.utu.fi/ZMUT.5836.

*Non-type material* (only diagnostic characters checked): UGANDA

• 18 ♀; Kibale National Park, Kanyawara; Tapani Hopkins leg.; ZMUThttp://mus.utu.fi/ZMUT.1259, http://mus.utu.fi/ZMUT.2057, http://mus.utu.fi/ZMUT.2203, http://mus.utu.fi/ZMUT.2358, http://mus.utu.fi/ZMUT.2627, http://mus.utu.fi/ZMUT.3199, http://mus.utu.fi/ZMUT.3649, http://mus.utu.fi/ZMUT.4866, http://mus.utu.fi/ZMUT.5596, http://mus.utu.fi/ZMUT.5647, http://mus.utu.fi/ZMUT.5679, http://mus.utu.fi/ZMUT.5712, http://mus.utu.fi/ZMUT.5717, http://mus.utu.fi/ZMUT.5736, http://mus.utu.fi/ZMUT.5803, http://mus.utu.fi/ZMUT.5804, http://mus.utu.fi/ZMUT.5805, http://mus.utu.fi/ZMUT.5835

• 20 ♂; Kibale National Park, Kanyawara; Tapani Hopkins leg.; ZMUThttp://mus.utu.fi/ZMUT.1350, http://mus.utu.fi/ZMUT.1355, http://mus.utu.fi/ZMUT.1401, http://mus.utu.fi/ZMUT.1765, http://mus.utu.fi/ZMUT.2645, http://mus.utu.fi/ZMUT.2657, http://mus.utu.fi/ZMUT.2803, http://mus.utu.fi/ZMUT.3076, http://mus.utu.fi/ZMUT.3081, http://mus.utu.fi/ZMUT.3087, http://mus.utu.fi/ZMUT.3097, http://mus.utu.fi/ZMUT.3761, http://mus.utu.fi/ZMUT.3876, http://mus.utu.fi/ZMUT.4990, http://mus.utu.fi/ZMUT.5143, http://mus.utu.fi/ZMUT.5609, http://mus.utu.fi/ZMUT.5768, http://mus.utu.fi/ZMUT.5787, http://mus.utu.fi/ZMUT.5831, http://mus.utu.fi/ZMUT.6033.

**Figures 39–45.**
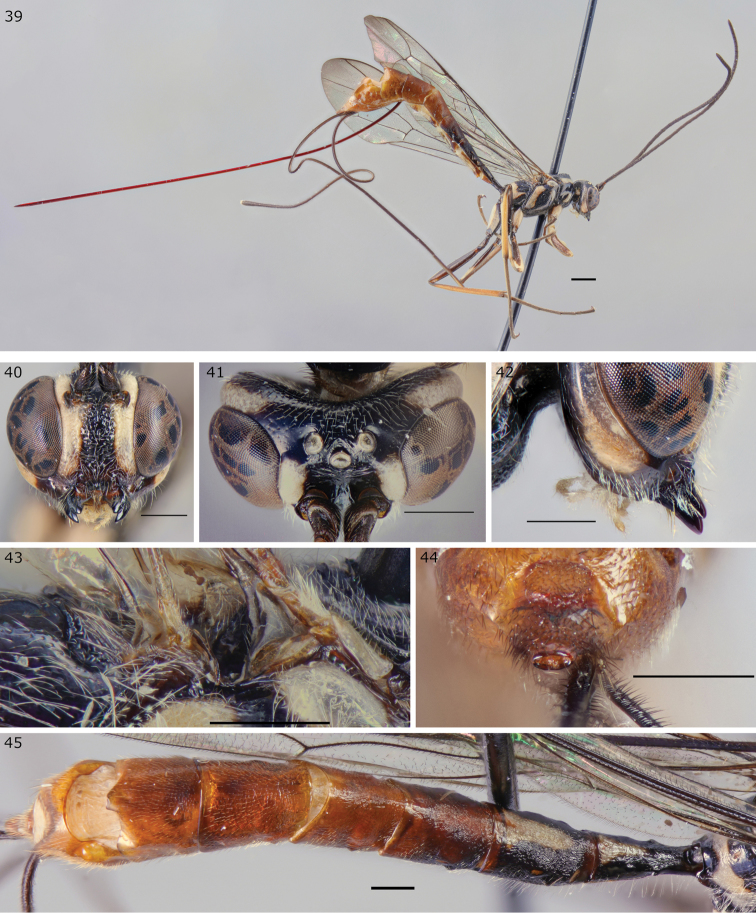
*Epirhyssa quagga* female (holotype http://mus.utu.fi/ZMUT.5788), a new species from Uganda. **39** Habitus **40** face and clypeus **41** frons **42** hypostomal flange **43** mesopleuron dorsal margin **44** apical horn of metasoma **45** tergites 1–7. Scale bars: 0.5 mm (**40–45**), 1 mm (**39**).

**Known material**: 46 specimens (23 ♀, 23 ♂, Ugandan specimens, data above).

###### Diagnosis.

This species can be distinguished from other Afrotropical Rhyssinae by the distinctive pattern of striation on the frons and the densely striate tergite 3. No other species has the same colour pattern.

***Head***: frons without median carinae, without lateral carinae; hypostomal carina raised into a low flange, its height slightly less than or equivalent to the maximum width of the second maxillary palp segment.

***Mesosoma***: subalar prominence without a lateral flange; mesopleuron without a flange along the dorsal margin; epicnemial carina reaches the approximate height of the mesopleural pit.

***Metasoma***: tip of apical horn elliptical in posterior view; tergite 3 densely striate.

###### Description (female).

Body length 8.5 mm–14.3 mm (holotype 12.3 mm).

***Head***: Frons without clear median carinae, without lateral carinae, with faint median rugae that fan out towards ocelli. Occipital carina interrupted dorsally. Hypostomal carina raised into a low flange, its height slightly less than or equivalent to maximum width of second maxillary palp segment. Face densely and deeply punctate. Clypeus with little or no punctation, with a median apical tubercle. Antenna with 30–33 flagellar segments (32 in holotype).

***Mesosoma***: Subalar prominence without a lateral flange. Mesopleuron without a flange along dorsal margin. Epicnemial carina reaches approximate height of mesopleural pit. Fore wing with 2m-cu varying from clearly distal to opposite rs-m.

***Metasoma***: Tip of apical horn elliptical in posterior view. Tergites 1–5 with dense, light, predominantly longitudinal striation and punctation, 6–7 smoother and more pubescent, tergite 1 1.9–2.4 times as long as apically wide (1.9 in holotype).

***Colour***: General colour mottled black and white with metasoma orange testaceous from tergite 3 onwards. Hind tibia dark brown. Antennae black. Ovipositor sheaths black to dark testaceous. Wings hyaline.

***Variation***: Colour of the dark patches of the legs varies from entirely black to testaceous, the black and white metasomal colour extends onto tergite 3 in some individuals.

**Male.**

Similar to female. Body length 7.9 mm–10.4 mm. T1 3.2–3.6. Antenna with 29–33 flagellar segments. Males are smaller than females on average.

###### Etymology.

Refers to the colour pattern which is reminiscent of the plains zebra, especially its extinct subspecies, the quagga.

###### Distribution.

Uganda.

###### Biology.

In Uganda, this species was most abundantly caught during the dry season (Hopkins et al. 2019b). It has not been caught outside the forest. It appears to be attracted to decaying wood (although the sample size is too small for statistical significance).

##### Epirhyssa shaka

Taxon classificationAnimaliaHymenopteraIchneumonidae

Rousse & van Noort, 2014

A6876924-1D85-54F2-9137-0068EA7EE919

Figs 46–49

###### Material examined.

**Type material**: SOUTH AFRICA:

• 1 ♀, holotype; Natal, 2831 Dd Umlalazi Nat. Res., 1.5 km E of Mtunzini; 28°57'S, 31°45'E; Nov. 1978; R. M. Miller leg.; indigenous forest; Malaise trap; NMSA.

**Known material**: One specimen (1 ♀, see Rousse and van Noort 2014, data above).

###### Diagnosis.

This species can be distinguished from other Afrotropical Rhyssinae by the combination of a low hypostomal flange, an elliptical apical horn of the metasoma, and a punctate (over 50% of surface) tergite 3.

***Head***: frons with median carinae converging before continuing towards median ocellus, without lateral carinae; hypostomal carina raised into a low flange, its height slightly less than or equivalent to the maximum width of the second maxillary palp segment.

***Mesosoma***: subalar prominence without a lateral flange; mesopleuron without a flange along the dorsal margin; epicnemial carina reaches the approximate height of the mesopleural pit.

***Metasoma***: tip of apical horn elliptical in posterior view; tergite 3 punctate.

###### Distribution.

South Africa (KwaZulu-Natal).

**Figures 46–49.**
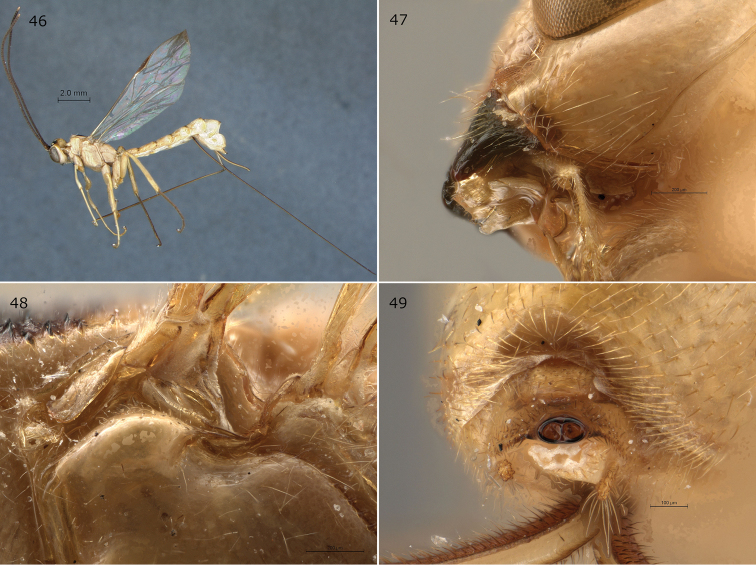
*Epirhyssa shaka* female (holotype). This species was not found in Uganda. **46** Habitus **47** hypostomal flange **48** mesopleuron dorsal margin **49** apical horn of metasoma. Figure **46** is from van Noort (2019).

##### Epirhyssa tombeaodiba

Taxon classificationAnimaliaHymenopteraIchneumonidae

Rousse & van Noort, 2014

94BAA882-EAC4-5CE6-ADB0-78992535B321

Figs 50–56

###### Material examined.

**Non-type material**: UGANDA:

• 1 ♀; Kibale National Park, Kanyawara, Site K30, Malaise trap K30T2; 0.5566N, 30.3633E (WGS84); alt. 1490 m (GPS, WGS84); 26 Aug. 2015–9 Sep. 2015; Tapani Hopkins leg.; ZMUThttp://mus.utu.fi/ZMUT.1273

• 1 ♀; same data as preceding; Site K15, Malaise trap K15T1; 0.5850N, 30.3641E (WGS84); alt. 1490 m (GPS, WGS84); 20 Apr. 2015–4 May 2015; ZMUThttp://mus.utu.fi/ZMUT.1333

• 1 ♀; same data as preceding; Site HILL, Malaise trap HILLT2; 0.5478N, 30.3619E (WGS84); alt. 1510 m (GPS, WGS84); 4 Jun. 2015–18 Jun. 2015; ZMUThttp://mus.utu.fi/ZMUT.3234

• 1 ♀; same data as preceding; Site K30S, Malaise trap K30ST3; 0.5378N, 30.3777E (WGS84); alt. 1480 m (GPS, WGS84); 19 May 2015–2 Jun. 2015; ZMUThttp://mus.utu.fi/ZMUT.5628

• 1 ♀; same data as preceding; Site K30, Malaise trap K30T3; 0.5590N, 30.3617E (WGS84); alt. 1540 m (GPS, WGS84); 14 Jul. 2015–28 Jul. 2015; ZMUThttp://mus.utu.fi/ZMUT.5663

• 1 ♀; same data as preceding; Site R93, Malaise trap R93T2; 0.5654N, 30.3593E (WGS84); alt. 1510 m (GPS, WGS84); 9 Mar. 2015–23 Mar. 2015; ZMUThttp://mus.utu.fi/ZMUT.5705.

**Known material**: 10 specimens (6 Ugandan, 4 other):

6 ♀; Ugandan specimens, data above and also in supplementary material (Hopkins et al. 2019c).

1 ♀, holotype; see Rousse and van Noort (2014); Cameroon, Nkoemvon [02°48'N 11°08'E]; 16 Mar. 1980–4 May 1980; “Ms. D. Jackson”; NHMUK.

1 ♀, paratype; see Rousse and van Noort (2014), same data as holotype.

1 ♀, paratype; see Rousse and van Noort (2014), same data as holotype except 30 Mar. 1980–19 Apr. 1980.

1 ♀, paratype; see Rousse and van Noort (2014), same data as holotype except Korup; 1981; “Mrs D. Jackson”

###### Diagnosis.

This species can be distinguished from other Afrotropical Rhyssinae by the combination of a half-elliptical apical horn of the metasoma and a punctate (over 50% of surface) tergite 3. *Epirhyssa uelensis* is also predominantly yellow with black spots, but its subalar prominence has a lateral flange.

***Head***: frons with weak median carinae converging before continuing towards median ocellus, without lateral carinae; hypostomal carina raised into a low flange, its height slightly less than or equivalent to the maximum width of the second maxillary palp segment.

***Mesosoma***: subalar prominence without a lateral flange; mesopleuron without a flange along the dorsal margin; epicnemial carina reaches the approximate height of the mesopleural pit.

***Metasoma***: tip of apical horn half-elliptical in posterior view; tergite 3 densely punctate.

###### Additional or updated characters.

Apart from the diagnosis, we provide the following additional or updated character traits to the description in Rousse and van Noort (2014).

**Female.**

Body length 7.2 mm–12.7 mm. Frons without rugae or with faint lateral rugae curving towards lateral ocelli. Face punctate or transversely rugulose-punctate. Clypeus longitudinally strigose and sparsely punctate. Antenna with 28–29 flagellar segments. Tergites 1 mostly smooth, 2–7 punctate (2 sometimes only punctate laterally), anterior margins of 5–6 often striate, tergite 1 1.2–1.5 times as long as apically wide. The Ugandan specimens have black anterior median spots on tergites 1–7 (ranging from very small on tergite 1 to reaching posterior margin on tergite 7), not just on tergites 4–7.

###### Distribution.

Cameroon. New record: Uganda.

**Figures 50–56.**
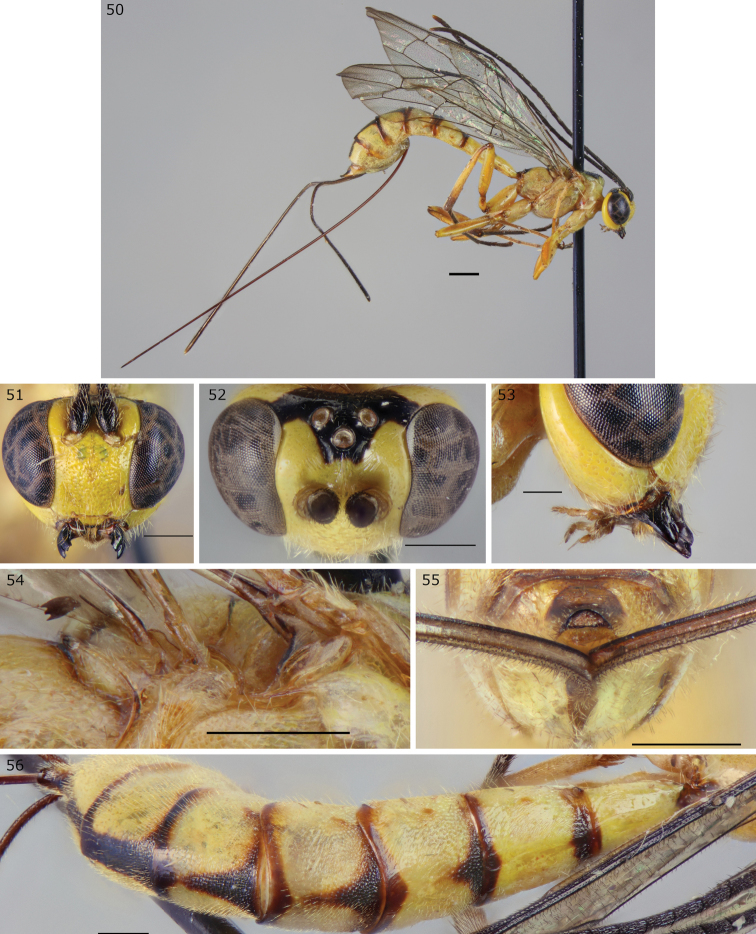
*Epirhyssa tombeaodiba* female (http://mus.utu.fi/ZMUT.5663, **52**: http://mus.utu.fi/ZMUT.3234), a species found in Uganda. **50** Habitus **51** face and clypeus **52** frons **53** hypostomal flange **54** mesopleuron dorsal margin **55** apical horn of metasoma **56** tergites 1–7. Scale bars: 0.5 mm (**51–56**), 1 mm (**50**).

##### Epirhyssa uelensis

Taxon classificationAnimaliaHymenopteraIchneumonidae

Benoit, 1951

7E4997D9-C669-5140-B122-21AF66A1328B

Figs 57–63 , 64–67

Epirhyssa gavinbroadi Rousse & van Noort, 2014, syn. nov.

###### Material examined.

**Type material**: CAMEROON:

• 1 ♀, holotype of *E. gavinbroadi*; Nkoemvon [Nko’emvon]; 02°48'N, 11°08'E; 16 Mar. 1980–4 May 1980; NHMUK type number HYM 3b.2832.

**Non-type material**: UGANDA

• 1 ♀; Kibale National Park, Kanyawara, Site K31, Malaise trap K31T4; 0.5362N, 30.3486E (WGS84); alt. 1460 m (GPS, WGS84); 16 Jul. 2015–30 Jul. 2015; Tapani Hopkins leg.; ZMUThttp://mus.utu.fi/ZMUT.2292

• 1 ♀; same data as preceding; Site K30, Malaise trap K30T3; 0.5590N, 30.3617E (WGS84); alt. 1540 m (GPS, WGS84); 21 Apr. 2015–5 May 2015; ZMUThttp://mus.utu.fi/ZMUT.2520

• 1 ♀; same data as preceding; Site K31, Malaise trap K31T4; 0.5362N, 30.3486E (WGS84); alt. 1460 m (GPS, WGS84); 30 Jan. 2015–13 Feb. 2015; ZMUThttp://mus.utu.fi/ZMUT.2878

• 1 ♀; same data as preceding; Site K13, Malaise trap K13T1; 0.5932N, 30.3598E (WGS84); alt. 1460 m (GPS, WGS84); 13 Jul. 2015–27 Jul. 2015; ZMUThttp://mus.utu.fi/ZMUT.5665

• 1 ♀; same data as preceding; Site K30, Malaise trap K30T2; 0.5566N, 30.3633E (WGS84); alt. 1490 m (GPS, WGS84); 2 Dec. 2014–15 Dec. 2014; ZMUThttp://mus.utu.fi/ZMUT.5689

• 1 ♀; same data as preceding; Site CC, Malaise trap CCT1; 0.5497N, 30.3673E (WGS84); alt. 1450 m (GPS, WGS84); 27 Jan. 2015–10 Feb. 2015; ZMUThttp://mus.utu.fi/ZMUT.5732

• 1 ♀; same data as preceding; 24 Feb. 2015–10 Mar. 2015; ZMUThttp://mus.utu.fi/ZMUT.5739

• 1 ♀; same data as preceding; Site K30S, Malaise trap K30ST4; 0.5414N, 30.3755E (WGS84); alt. 1420 m (GPS, WGS84); 30 Jun. 2015–14 Jul. 2015; ZMUThttp://mus.utu.fi/ZMUT.5844

• 1 ♂; Kibale National Park, Kanyawara, Site R93, Malaise trap R93T2; 0.5654N, 30.3593E (WGS84); alt. 1510 m (GPS, WGS84); 20 May 2015–1 Jun. 2015; Tapani Hopkins leg.; ZMUThttp://mus.utu.fi/ZMUT.5765

• 1 ♂; same data as preceding; Site K13, Malaise trap K13T1; 0.5932N, 30.3598E (WGS84); alt. 1460 m (GPS, WGS84); 4 May 2015–20 May 2015; ZMUThttp://mus.utu.fi/ZMUT.5807.

**Non-type material** (only diagnostic characters checked): UGANDA:

• 150 ♀; Kibale National Park, Kanyawara; Tapani Hopkins leg.; ZMUThttp://mus.utu.fi/ZMUT.1265, http://mus.utu.fi/ZMUT.1266, http://mus.utu.fi/ZMUT.1267, http://mus.utu.fi/ZMUT.1268, http://mus.utu.fi/ZMUT.1272, http://mus.utu.fi/ZMUT.1334, http://mus.utu.fi/ZMUT.1366, http://mus.utu.fi/ZMUT.1367, http://mus.utu.fi/ZMUT.1375, http://mus.utu.fi/ZMUT.1499, http://mus.utu.fi/ZMUT.1673, http://mus.utu.fi/ZMUT.1677, http://mus.utu.fi/ZMUT.1763, http://mus.utu.fi/ZMUT.1764, http://mus.utu.fi/ZMUT.1807, http://mus.utu.fi/ZMUT.2015, http://mus.utu.fi/ZMUT.2152, http://mus.utu.fi/ZMUT.2162, http://mus.utu.fi/ZMUT.2165, http://mus.utu.fi/ZMUT.2172, http://mus.utu.fi/ZMUT.2299, http://mus.utu.fi/ZMUT.2317, http://mus.utu.fi/ZMUT.2340, http://mus.utu.fi/ZMUT.2361, http://mus.utu.fi/ZMUT.2362, http://mus.utu.fi/ZMUT.2364, http://mus.utu.fi/ZMUT.2473, http://mus.utu.fi/ZMUT.2573, http://mus.utu.fi/ZMUT.2640, http://mus.utu.fi/ZMUT.2642, http://mus.utu.fi/ZMUT.2643, http://mus.utu.fi/ZMUT.2644, http://mus.utu.fi/ZMUT.2646, http://mus.utu.fi/ZMUT.2648, http://mus.utu.fi/ZMUT.2887, http://mus.utu.fi/ZMUT.2910, http://mus.utu.fi/ZMUT.3022, http://mus.utu.fi/ZMUT.3090, http://mus.utu.fi/ZMUT.3091, http://mus.utu.fi/ZMUT.3093, http://mus.utu.fi/ZMUT.3094, http://mus.utu.fi/ZMUT.3096, http://mus.utu.fi/ZMUT.3102, http://mus.utu.fi/ZMUT.3106, http://mus.utu.fi/ZMUT.3135, http://mus.utu.fi/ZMUT.3225, http://mus.utu.fi/ZMUT.3259, http://mus.utu.fi/ZMUT.3262, http://mus.utu.fi/ZMUT.3334, http://mus.utu.fi/ZMUT.3438, http://mus.utu.fi/ZMUT.3452, http://mus.utu.fi/ZMUT.3619, http://mus.utu.fi/ZMUT.3698, http://mus.utu.fi/ZMUT.3743, http://mus.utu.fi/ZMUT.3745, http://mus.utu.fi/ZMUT.3747, http://mus.utu.fi/ZMUT.3947, http://mus.utu.fi/ZMUT.4424, http://mus.utu.fi/ZMUT.4512, http://mus.utu.fi/ZMUT.4517, http://mus.utu.fi/ZMUT.5086, http://mus.utu.fi/ZMUT.5315, http://mus.utu.fi/ZMUT.5381, http://mus.utu.fi/ZMUT.5542, http://mus.utu.fi/ZMUT.5589, http://mus.utu.fi/ZMUT.5593, http://mus.utu.fi/ZMUT.5597, http://mus.utu.fi/ZMUT.5601, http://mus.utu.fi/ZMUT.5605, http://mus.utu.fi/ZMUT.5606, http://mus.utu.fi/ZMUT.5607, http://mus.utu.fi/ZMUT.5608, http://mus.utu.fi/ZMUT.5613, http://mus.utu.fi/ZMUT.5614, http://mus.utu.fi/ZMUT.5616, http://mus.utu.fi/ZMUT.5618, http://mus.utu.fi/ZMUT.5619, http://mus.utu.fi/ZMUT.5620, http://mus.utu.fi/ZMUT.5623, http://mus.utu.fi/ZMUT.5625, http://mus.utu.fi/ZMUT.5629, http://mus.utu.fi/ZMUT.5631, http://mus.utu.fi/ZMUT.5632, http://mus.utu.fi/ZMUT.5635, http://mus.utu.fi/ZMUT.5637, http://mus.utu.fi/ZMUT.5641, http://mus.utu.fi/ZMUT.5642, http://mus.utu.fi/ZMUT.5643, http://mus.utu.fi/ZMUT.5644, http://mus.utu.fi/ZMUT.5646, http://mus.utu.fi/ZMUT.5648, http://mus.utu.fi/ZMUT.5650, http://mus.utu.fi/ZMUT.5653, http://mus.utu.fi/ZMUT.5655, http://mus.utu.fi/ZMUT.5657, http://mus.utu.fi/ZMUT.5662, http://mus.utu.fi/ZMUT.5668, http://mus.utu.fi/ZMUT.5669, http://mus.utu.fi/ZMUT.5670, http://mus.utu.fi/ZMUT.5673, http://mus.utu.fi/ZMUT.5678, http://mus.utu.fi/ZMUT.5681, http://mus.utu.fi/ZMUT.5683, http://mus.utu.fi/ZMUT.5684, http://mus.utu.fi/ZMUT.5687, http://mus.utu.fi/ZMUT.5688, http://mus.utu.fi/ZMUT.5690, http://mus.utu.fi/ZMUT.5692, http://mus.utu.fi/ZMUT.5693, http://mus.utu.fi/ZMUT.5694, http://mus.utu.fi/ZMUT.5698, http://mus.utu.fi/ZMUT.5699, http://mus.utu.fi/ZMUT.5702, http://mus.utu.fi/ZMUT.5708, http://mus.utu.fi/ZMUT.5709, http://mus.utu.fi/ZMUT.5719, http://mus.utu.fi/ZMUT.5722, http://mus.utu.fi/ZMUT.5725, http://mus.utu.fi/ZMUT.5726, http://mus.utu.fi/ZMUT.5733, http://mus.utu.fi/ZMUT.5734, http://mus.utu.fi/ZMUT.5740, http://mus.utu.fi/ZMUT.5748, http://mus.utu.fi/ZMUT.5750, http://mus.utu.fi/ZMUT.5754, http://mus.utu.fi/ZMUT.5755, http://mus.utu.fi/ZMUT.5757, http://mus.utu.fi/ZMUT.5762, http://mus.utu.fi/ZMUT.5763, http://mus.utu.fi/ZMUT.5764, http://mus.utu.fi/ZMUT.5772, http://mus.utu.fi/ZMUT.5774, http://mus.utu.fi/ZMUT.5775, http://mus.utu.fi/ZMUT.5778, http://mus.utu.fi/ZMUT.5780, http://mus.utu.fi/ZMUT.5794, http://mus.utu.fi/ZMUT.5797, http://mus.utu.fi/ZMUT.5806, http://mus.utu.fi/ZMUT.5808, http://mus.utu.fi/ZMUT.5811, http://mus.utu.fi/ZMUT.5816, http://mus.utu.fi/ZMUT.5817, http://mus.utu.fi/ZMUT.5821, http://mus.utu.fi/ZMUT.5823, http://mus.utu.fi/ZMUT.5824, http://mus.utu.fi/ZMUT.5825, http://mus.utu.fi/ZMUT.5829, http://mus.utu.fi/ZMUT.5834, http://mus.utu.fi/ZMUT.5845, http://mus.utu.fi/ZMUT.5846.

**Known material**: 167 specimens (160 Ugandan, 7 other):

158 ♀, 2 ♂; Ugandan specimens, data above and also in supplementary material (Hopkins et al. 2019c).

1 ♀, holotype of *E. gavinbroadi*; see Rousse and van Noort (2014), data above in material examined.

1 ♀, holotype of *E. uelensis*; see Rousse and van Noort (2014); Democratic Republic of Congo, Haut-Uele, Paulis [Isiro, 03°28'N 25°43'E]; Dec. 1947; “P.L.G. Benoit”; MRAC.

1 ♂, paratype of *E. uelensis*; see Rousse and van Noort (2014); Democratic Republic of Congo, Bambesa; Jul. 1933; “H.J. Bredo”; MRAC.

1 ♀; see Rousse and van Noort (2014); Cameroon, Nkoemvon; 30 Mar. 1980–19 Apr. 1980; “Ms. D. Jackson”; NHMUK.

2 ♀; see Rousse and van Noort (2014), same data as previous except 13 Jul. 1980–4 Aug. 1980.

1 ♀; see Rousse and van Noort (2014), same data as previous except Oct. 1980–Nov. 1980.

**Figures 57–63.**
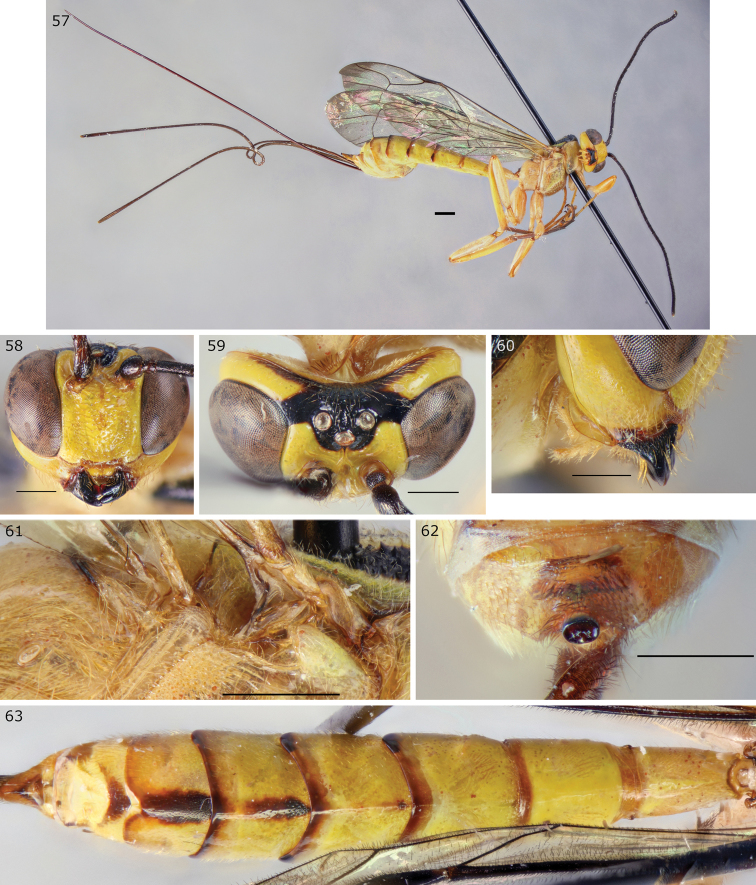
*Epirhyssa uelensis* female (http://mus.utu.fi/ZMUT.2520), a species found in Uganda. **57** Habitus **58** face and clypeus **59** frons **60** hypostomal flange **61** mesopleuron dorsal margin **62** apical horn of metasoma **63** tergites 1–7. Scale bars: 0.5 mm (**58–63**), 1 mm (**57**).

###### Diagnosis.

This species can be distinguished from other Afrotropical Rhyssinae by its subalar prominence having a lateral flange. *Epirhyssa tombeaodiba* is also predominantly yellow with black spots but lacks the flange.

***Head***: frons with median carinae converging before continuing towards median ocellus, with lateral carinae curving towards lateral ocelli; hypostomal carina raised into an elevated flange, its height greater than the maximum width of the second maxillary palp segment.

***Mesosoma***: subalar prominence with a lateral flange; mesopleuron with a raised flange along the dorsal margin; epicnemial carina reaches the approximate height of the mesopleural pit.

***Metasoma***: tip of apical horn elliptical in posterior view; tergite 3 mostly smooth.

###### Additional or updated characters.

Apart from the diagnosis, we provide the following additional or updated character traits to the description in Rousse and van Noort (2014).

**Female.** Body length 9.8 mm–15.7 mm (15.7 mm *E. gavinbroadi* holotype, 9.8 mm–15.1 mm other specimens). Frons with more or less distinct rugae parallel to the lateral carinae, often as distinct as the lateral carinae. Clypeus sparsely punctate or smooth. Antenna with 30–35+ flagellar segments (30–33 in Ugandan specimens, at least 35 in *E. gavinbroadi* type, 30–34 in other specimens). Fore wing with 2m-cu varying from clearly distal to only just distal to rs-m. Tip of apical horn with an extra dorsal projection in one aberrant female (http://mus.utu.fi/ZMUT.5844). Tergites mostly smooth, except with varied amount of punctation on tergites 3–7 (anteriorly punctate 3–6 in many Ugandan specimens, punctate 3–6 in most non-Ugandan specimens, punctate apex of 5 and 6–7 in *E. gavinbroadi* holotype), anterior margins of 3–5 or 3–6 often striate, tergite 1 1.2–1.7 times as long as apically wide (1.7 in *E. gavinbroadi* holotype, 1.2–1.5 in other specimens). The Ugandan specimens have black posterior margins of tergites 2–7 (sometimes faint or patchy, especially on anterior tergites) and median stripes on 4–7 (strongest on 5–6). Non-Ugandan specimens sometimes have black median stripes on 5–7, or 3–6 in the *E. gavinbroadi* holotype.

**Male.** Body length 8.5 mm–8.7 mm. Antenna with 30 flagellar segments. Tergites 4–7 faintly pubescent, tergite anterior margins smooth, or lightly striate and punctate, tergite 1 1.5–2.0 times as long as apically wide. The two Ugandan males are smaller than females on average.

###### Distribution.

Democratic Republic of Congo, Cameroon. New record: Uganda.

###### Biology.

In Uganda, this species was most abundantly caught in primary forest near decaying wood, during the dry season (Hopkins et al. 2019b). It has not been caught outside the forest.

###### Remarks.

This species is quite variable, but we are unable to find morphological characters that would reliably split it into more than one species. We propose that *Epirhyssa gavinbroadi* (of which there is only one specimen) is a synonym of *E. uelensis*. It was mainly distinguished from *E. uelensis* by the punctate clypeus and slender tergite 1, but the Ugandan specimens generally have a punctate clypeus and a stout tergite 1. These two characters also vary considerably in the Ugandan specimens and seem to represent intraspecific variation. The Ugandan material has a very skewed sex ratio with 158 female specimens collected and only two males.

**Figures 64–67.**
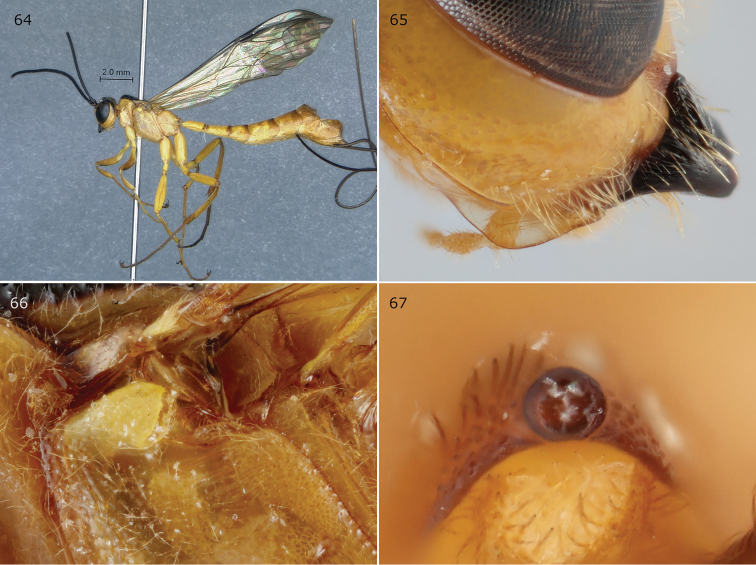
*Epirhyssa gavinbroadi* female (holotype). We treat this species as a synonym of *E. uelensis* in this work. **64** Habitus **65** hypostomal flange **66** mesopleuron dorsal margin **67** apical horn of metasoma. Figure **64** is from van Noort (2019).

##### Epirhyssa villemantae

Taxon classificationAnimaliaHymenopteraIchneumonidae

Rousse & van Noort, 2014

C8AD2480-43A0-52F4-9C2B-51A04A3F54DA

Figs 68–71

###### Material examined.

**Type material**: NIGERIA:

• 1 ♀, holotype; “Ilorin Prov.” [06°48'N, 05°18'E]; “18 Jun. 192” [18 Jun 1921?]; “De. G.W.S. Macfie” leg.; “Pres. by Imp. Bur. Ent. 1921–129”; NHMUK.

**Known material**: One specimen (1 ♀, see Rousse and van Noort 2014, data above).

###### Diagnosis.

This species can be distinguished from other Afrotropical Rhyssinae by the combination of lateral carinae on the frons and the absence of a raised flange on the dorsal margin of the mesopleuron. No other species has the same colour pattern.

***Head***: frons with median carinae diverging towards ocelli, with lateral carinae curving towards lateral ocelli; hypostomal carina raised into an elevated flange, its height greater than the maximum width of the second maxillary palp segment.

***Mesosoma***: subalar prominence without a lateral flange; mesopleuron without a flange along the dorsal margin; epicnemial carina reaches the approximate height of the mesopleural pit.

***Metasoma***: tip of apical horn elliptical in posterior view; tergite 3 mostly smooth.

###### Distribution.

Nigeria.

**Figures 68–71.**
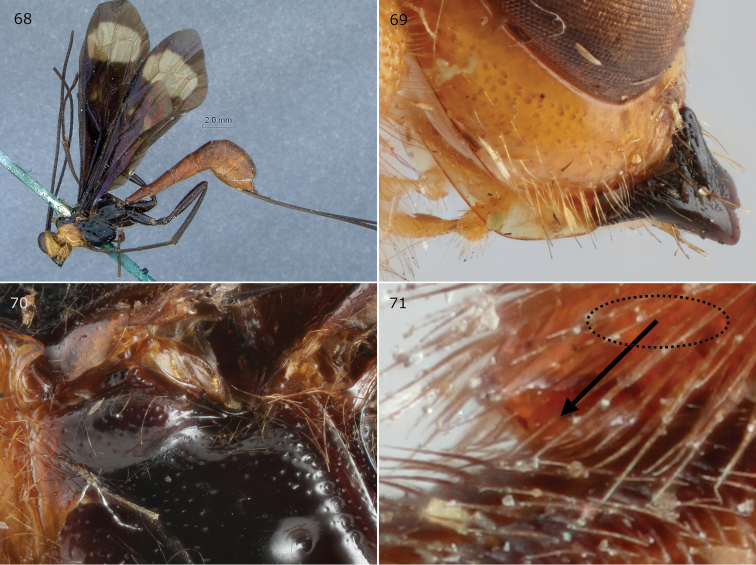
*Epirhyssa villemantae* female (holotype). This species was not found in Uganda. **68** Habitus **69** hypostomal flange **70** mesopleuron dorsal margin **71** apical horn of metasoma. Figure **68** is from van Noort (2019).

##### Genus Megarhyssa

Taxon classificationAnimaliaHymenopteraIchneumonidae

Ashmead, 1900

08CA33FC-BBEC-5FC1-8D5E-6CF0779A36D9

Thalessa Holmgren, 1859: 122.
Megalorhyssa Shulz, 1906: 115.
Eurhyssa Derksen, 1941: 721.

###### Diagnosis.

The genus *Megarhyssa* is easily recognised in the Afrotropical region by the presence of the fore wing areolet (vein 3rs-m is present), whereas vein 3rs-m is missing in *Epirhyssa*, the only other rhyssine genus found in the Afrotropical region.

*Megarhyssa* can be distinguished from other rhyssine genera by the presence of an areolet (cf. *Triancyra*), the lack of an anterior glymma on tergite 1 (cf. *Rhyssa*), the upper tooth not being subdivided (cf. *Myllenyxis*), tergite 1 having anterior lateral carinae (cf. *Cyrtorhyssa* which lacks carinae), the occipital carina joining the hypostomal carina some distance from the mandible base (cf. *Lytarmes*) and tergites 3–6 not being transversely, non-uniformly aciculate (cf. *Rhyssella*) (Baltazar 1964, Porter 2001). The genus includes the largest species of Rhyssinae.

###### Distribution.

**Afrotropical region**: Democratic Republic of Congo.

The genus is cosmopolitan with the largest number of species found in the Oriental and Palaearctic regions.

##### Megarhyssa babaulti

Taxon classificationAnimaliaHymenopteraIchneumonidae

Seyrig, 1937

B7534017-F63C-5CB3-91B3-3BCF6209247F

Figs 72–75

###### Material examined.

**Type material**: DEMOCRATIC REPUBLIC OF CONGO:

• 1 ♀, holotype; “reg. Lac Kivu”, “Kadjudju” [Kajuju, 02°09'S, 28°54'E]; 1932; MNHN EY8831.

**Known material**: One specimen (1 ♀, see Rousse and van Noort 2014, data above).

###### Diagnosis.

This species can be distinguished from other Afrotropical Rhyssinae by the presence of a closed areolet. No other species has the same colour pattern.

***Head***: frons with median carinae diverging towards ocelli, without lateral carinae; hypostomal carina raised into an elevated flange, its height slightly greater than the maximum width of the second maxillary palp segment.

***Mesosoma***: subalar prominence without a lateral flange; mesopleuron without a flange along the dorsal margin; epicnemial carina laterally absent.

***Metasoma***: tip of apical horn almost circular in posterior view; tergite 3 mostly smooth.

###### Distribution.

Democratic Republic of Congo.

**Figures 72–75.**
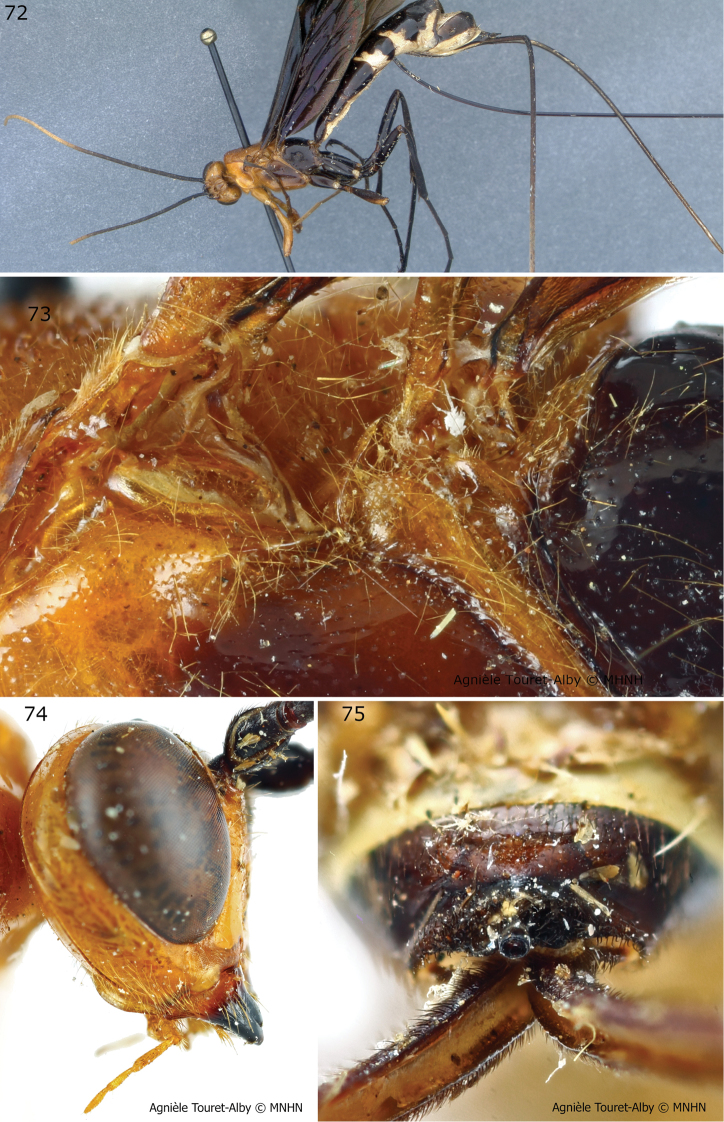
*Megarhyssa babaulti* female (holotype). This species was not found in Uganda. **72** Habitus **73** mesopleuron dorsal margin **74** hypostomal flange **75** apical horn of metasoma. Figure **72** is from van Noort (2019), **73–75** courtesy of MNHN (Agnièle Touret-Alby).

## Discussion

Sampling tropical ichneumonids for a year with numerous Malaise traps gave us an unprecedented sample size. We caught 456 individuals of the subfamily Rhyssinae, which in the Afrotropical region was earlier known from only 30 published specimen records (Rousse and van Noort 2014). This clearly demonstrates the advantage of extensive, long-term sampling in the tropics. Although the method is laborious, involving a full year of sampling and several years processing the samples, it provides much greater sample sizes than the more limited sampling which is often the norm due to logistical constraints (see Owen and Chanter 1970, van Noort et al. 2000, van Noort 2004). We were also able to obtain information on the phenology and habitat use of species, by linking to vegetation and weather data (Hopkins et al. 2019b).

Our results strongly support the idea that the Afrotropical region contains a large number of rhyssine species, most of which have simply not been discovered due to insufficient sampling (Rousse and van Noort 2014). Two of the six species at our site were new to science. New information was also obtained on, for example, the male of *Epirhyssa overlaeti* and the synonymy of *E. gavinbroadi* with *E. uelensis*. It is clear that sampling at other Afrotropical sites would reveal numerous new species, and considerably update our knowledge of described species. Further sampling would likely uncover more species even at our site; we caught only one individual of *E. johanna* sp. nov., for example, which demonstrates that our site is still under-sampled.

Our results also support the claim by Quicke (2012) that it is too early to draw conclusions on how ichneumonid species richness is distributed on our planet. Before this work, no rhyssines were known from our site despite our having sampled there before (Hopkins et al. 2018, SvN pers. obs.). After this work, the species count is six, and further sampling would likely discover more. In such circumstances, any attempt to compare the species richness of different sites may end up merely comparing sample sizes. Of two recent studies that made this attempt, one found the highest rhyssine and pimpline richness at Allpahuayo-Mishana in Amazonia, which may be due to the fact it is also one of the best sampled sites (Gómez et al. 2017). The other study showed that even well sampled sites are so under-sampled that species richness and sample size are interchangeable (Timms et al. 2016, although note that the authors interpret this result differently). Much more sampling, especially in the tropics, will be needed before we can draw conclusions on ichneumonid species richness distributions.

## Supplementary Material

XML Treatment for Epirhyssa

XML Treatment for Epirhyssa brianfisheri

XML Treatment for Epirhyssa ghesquierei

XML Treatment for Epirhyssa johanna

XML Treatment for Epirhyssa leroyi

XML Treatment for Epirhyssa maynei

XML Treatment for Epirhyssa migratoria

XML Treatment for Epirhyssa overlaeti

XML Treatment for Epirhyssa quagga

XML Treatment for Epirhyssa shaka

XML Treatment for Epirhyssa tombeaodiba

XML Treatment for Epirhyssa uelensis

XML Treatment for Epirhyssa villemantae

XML Treatment for Megarhyssa

XML Treatment for Megarhyssa babaulti

## References

[B1] Baltazar CR (1964) The genera of parasitic Hymenoptera in the Philippines, part 2. Pacific Insects 6: 53 pp.

[B2] Gaston KJ Gauld ID (1993) How many species of pimplines (Hymenoptera: Ichneumonidae) are there in Costa Rica? Journal of Tropical Ecology 9: 491–499. 10.1017/S0266467400007550

[B3] Gauld ID (1991) The Ichneumonidae of Costa Rica, 1. Memoirs of the American Entomological Institute 47: 1–589.

[B4] Gómez IC Sääksjärvi IE Mayhew PJ Pollet M Rey del Castillo C Nieves-Aldrey J-L Broad GR Roininen H Tuomisto H (2017) Variation in the species richness of parasitoid wasps (Ichneumonidae: Pimplinae and Rhyssinae) across sites on different continents. Insect Conservation and Diversity. 10.1111/icad.12281

[B5] Hopkins T Roininen H Sääksjärvi IE (2018) Assessing the species richness of Afrotropical ichneumonid wasps with randomly placed traps provides ecologically informative data. African Entomology 26: 350–359. 10.4001/003.026.0350

[B6] Hopkins T Roininen H Sääksjärvi IE (2019a) Uganda Malaise trapping 2014–2015 background data [Dataset]. Zenodo. 10.5281/zenodo.2225643

[B7] Hopkins T Roininen H Sääksjärvi IE (2019b) Extensive sampling reveals the phenology and habitat use of Afrotropical parasitoid wasps (Hymenoptera: Ichneumonidae: Rhyssinae). Royal Society Open Science 6: 190913. 10.1098/rsos.190913 PMC6731719 31598258

[B8] Hopkins T Roininen H van Noort S Broad GR Kaunisto K Sääksjärvi IE (2019c) Uganda Malaise trapping 2014–2015 Rhyssinae taxonomy data [Dataset]. Zenodo. 10.5281/zenodo.2554877

[B9] Janzen DH Pond CM (1975) A comparison, by sweep sampling, of the arthropod fauna of secondary vegetation in Michigan, England and Costa Rica. Transactions of the Royal Entomological Society of London 127: 33–50. 10.1111/j.1365-2311.1975.tb00551.x

[B10] Morrison G Auerbach M McCoy ED (1979) Anomalous Diversity of Tropical Parasitoids: A General Phenomenon? The American Naturalist 114: 303–307. 10.1086/283477

[B11] Owen DF Chanter DO (1970) Species Diversity and Seasonal Abundance in Tropical Ichneumonidae. Oikos 21: 142–144. 10.2307/3543849

[B12] Owen DF Owen J (1974) Species diversity in temperate and tropical Ichneumonidae. Nature 249: 583–584. 10.1038/249583a0

[B13] Porter CC (2001) New species and records of *Rhyssa* and *Rhyssella* (Hymenoptera: Ichneumonidae: Rhyssinae) from Florida and Central America. Insecta Mundi 15: 129–137.

[B14] Quicke DLJ (2012) We know too little about parasitoid wasp distributions to draw any conclusions about latitudinal trends in species richness, body size and biology. PLoS ONE 7: e32101. 10.1371/journal.pone.0032101 PMC3280234 22355411

[B15] Rousse P van Noort S (2014) A review of the Afrotropical Rhyssinae (Hymenoptera: Ichneumonidae) with the descriptions of five new species. European Journal of Taxonomy 91: 1–42. 10.5852/ejt.2014.91

[B16] Sääksjärvi IE Haataja S Neuvonen S Gauld ID Jussila R Salo J Burgos AM (2004) High local species richness of parasitic wasps (Hymenoptera: Ichneumonidae; Pimplinae and Rhyssinae) from the lowland rainforests of Peruvian Amazonia. Ecological Entomology 29: 735–743. 10.1111/j.0307-6946.2004.00656.x

[B17] Shapiro BA Pickering J (2000) Rainfall and parasitic wasp (Hymenoptera: Ichneumonoidea) activity in successional forest stages at Barro Colorado Nature Monument, Panama, and La Selva Biological Station, Costa Rica. Agricultural and Forest Entomology 2: 39–47. 10.1046/j.1461-9563.2000.00048.x

[B18] Timms LL Schwarzfeld M Sääksjärvi IE (2016) Extending understanding of latitudinal patterns in parasitoid wasp diversity. Insect Conservation and Diversity 9: 74–86. 10.1111/icad.12144

[B19] Townes H (1969) The genera of Ichneumonidae, Part 1. Memoirs of the American Entomological Institute 11: 1–300. 10.1007/BF02027741

[B20] Townes H Townes M (1973) A catalogue and reclassification of the Ethiopian Ichneumonidae. Memoirs of the American Entomological Institute 19: 1–416.

[B21] van Noort S (2004) Ichneumonid (Hymenoptera: Ichneumonoidea) diversity across an elevational gradient on Monts Doudou in Southwestern Gabon. California Academy of Sciences Memoir 28: 187–216.

[B22] van Noort S (2019) WaspWeb: Hymenoptera of the Afrotropical region. Available from: www.waspweb.org [accessed: July 2, 2019]

[B23] van Noort S Prinsloo GL Compton SG (2000) Hymenoptera, excluding Apoidea (Apiformes) & Formicidae (Insecta). Cimbebasia Memoir 9: 289–364.

[B24] Yu DS van Achterberg C Horstmann K (2016) Taxapad 2016, Ichneumonoidea 2015. Database on flash-drive. www.taxapad.com, Nepean.

